# Supplementary material for: Metaboverse enables automated discovery and visualization of diverse metabolic regulatory patterns
Source: Nat Cell Biol. 2023 Apr 3;25(4):616–25. doi: 10.1038/s41556-023-01117-9 (PMC10104781; doi:10.1038/s41556-023-01117-9)
Supplement: Supplementary file 1 — Supplementary Notes 1–3. [file 41556_2023_1117_MOESM1_ESM.pdf]

nature cell biology

Technical Report

https://doi.org/10.1038/s41556-023-01117-9

Metaboverse enables automated discovery and visualization of diverse metabolic regulatory patterns

In the format provided by the authors and unedited

Contents

Supplementary Note 1: A brief overview and benchmarking of representative metabolic network analysis tools **2**
Overview 2
General analysis approaches 2
Other approaches 2

Supplementary Note 2: Methods & algorithms **3**
Network curation 3
Collapsing reactions with missing expression or abundance values in user data 5
Regulatory pattern searches and sorting 6

Supplementary Note 3: Equations **7**
Pattern search algorithms 7
Reaction complex median 8
Reaction complex aggregated p-value 8

References **9**

Supplementary Note 1: A brief overview and benchmarking of representative metabolic network analysis tools

Overview

Several computational tools have risen to prominence to attempt to resolve issues in metabolic data analysis and interpretation. However, many of these tools have limitations, such as relying solely on pathway-level analysis, searching only for a specific metabolic pattern, non-automated pattern recognition, and/or proprietary software restrictions. We have highlighted four such representative and popular tools for their respective properties, though many more exist [1]. Although, at the outset, we want to emphasize that these tools are unable to identify key patterns that Metaboverse facilely discovers and are verifiable, as we detail in this work. For an in-depth discussion of other metabolomics and metabolism computational tools, we refer readers to the resource paper, [2]. For the outputs generated through this benchmarking, see `https://github.com/Metaboverse/Metaboverse-manuscript/tree/main/data/benchmarking`.

General analysis approaches

- **Set Enrichment Analysis**: Related to the analysis of the enrichment of sets of significantly changed analytes (i.e., metabolite, protein, or gene measurements), for extracting interesting information. Perhaps among the most popular tools for metabolism-related set enrichment analysis are MetaboAnalyst [3,4] and Metascape [5]. While network visualization is available, it primarily focuses on interaction networks, and its ability to extract regulatory information is limited, particularly in an automated fashion. Set enrichment analysis also misses how specific sub-network components may interact with each other. For example, the data are generalized such that the enrichment of two network-adjacent metabolites does not necessarily influence the analysis. Additionally, the number of metabolites measured, which will vary dataset to dataset in metabolomics, can dramatically influence enrichment results [6].
- **General Purpose Network Analysis**: The most popular (with $>$ 30,000 citations) general purpose network visualization and analysis suite is Cytoscape [7]. One plug-in for Cytoscape that focuses on metabolic data is MetScape, but this tool is generally limited to pathway enrichment, correlation networks, and data visualization. MetScape does not integrate approaches to identify regulatory mechanisms at the reaction-level within the data [7–10].
- **Metabolic Network Analysis**: MetExplore focuses on the curation of networks and is particularly useful for collaborative annotation of emerging models of organisms with incomplete metabolic network curations. Additionally, MetExplore can layer experimental data on the metabolic network for visualization [11,12]. A companion tool to MetExplore is MetExploreViz, which enables interactive and flexible visualization of omics data on metabolic networks [13]. Reactome, which our tool uses for the curation of biological networks, also offers analytical tools for user data, but relies on set enrichment or manual methods for identifying patterns [14–16]. While all have their respective utility, there is a pressing need for tools that integrate these features and automate pattern and trend detection across metabolic networks to extract regulatory and other features from data.
- **Metabolic Modeling**: A variety of tools exist that attempt to provide deeper analysis of metabolism-related data from a reaction network perspective. Recently, Compass was developed and used to predict reaction flux and metabolic state from transcriptomic data [17]. This tool provides exciting potential in the development of methods that get closer to making metabolic mechanistic predictions directly from user data. However, Compass is currently limited to processing transcriptomics data, requires computational background of the user, utilizes proprietary software dependencies which can complicate installation and usage of Compass, and does not currently provide a visual interface for exploring relevant patterns that are identified by the software. Additionally, the various layers of regulation between transcription and enzymatic behavior and metabolite flux may yet confound our ability to accurately predict reaction behavior across biological scenarios.

Other approaches

Additional tools of note include the activity motif [18], reporter reaction [19,20], and metabolic network segmentation (MNS) [21] methodologies. Previous work by Checkik, et al. introduced the concept of an activity motif, where a network pattern was based on the expression characteristics of sequential components in a signaling cascade [18]. Reporter reactions were similar in concept to activity motifs, but focused on identifying a singular pattern across all metabolic reactions generally based on the sum of z-scores. This mathematical approach thereby infers with potential reaction directionality. However, both activity motifs and reporter reactions only identify a single type of pattern, lack a visual component to their analysis, and to our knowledge do not have public, working code.

MNS overcomes some of these particular challenges, as it has a working code-base and outputs files that can be input into Cytoscape for visualization [7,21]. MNS is written in MATLAB, however, so its operation requires a MATLAB license. Ingenuity Pathway Analysis (IPA) similarly enables other methods of general pattern analysis, specifically referred to as “Upstream regulators” and “Causal networks”, given multi-omics data with more integrated visualization options. However, the code is closed source. IPA is also limited to analyzing human, rat, and mouse data and networks, thereby limiting its flexibility. Importantly, all of these tools were unable to identify the key reaction patterns discussed within this manuscript (Supplementary Table 1).

Supplementary Note 2: Methods & algorithms

Network curation

After the relevant information is parsed from each organism's relevant records, the global network is propagated using the NetworkX framework [22] to generate nodes for each reaction and reaction component and edges connecting components to the appropriate reactions. In some cases, a separate ID is used to generate two nodes for the same metabolite within two separate compartments to aid in downstream visualization; however, user data for the given entity would be mapped to both nodes.

After the network is curated for the user-specified organism, each node's degree (or magnitude of edges or connections) is determined to aid in the user's downstream ability to avoid visualizing high-degree components, such as a proton or water, on the metabolic network, which can lead to visual network entanglement and cluttering and a decrease in computational performance [23]. The user may also choose to add metabolites or other components to a blocklist, which will lead to these entities being ignored during analysis and visualization. The resulting graph template is output as a JSON-formatted `template.mvrs` file. A reaction neighbors JSON-formatted file is also output with the file suffix, `.nbdb`. These files, along with the initial `.mvdb` file are curated for each available organism with each release of Metaboverse to decrease back-end processing time on the user's machine. These files are hosted at `https://rutter.chpc.utah.edu/Metaboverse`.

Algorithm 1. `mvdb` database structure.

```
├─ Reactome species ID
├─ Reactome database version
├─ Curation date
├─ metaboverse-cli curation date
├─ Reactome database version
├─ Reactome database date
├─ pathway database
│  └─ for every pathway ID
│     ├─ pathway ID
│     ├─ Reactome ID
│     ├─ common name
│     └─ list of reactions
├─ reaction database
│  └─ for every reaction ID
│     ├─ reaction ID
│     ├─ Reactome ID
│     ├─ common name
│     ├─ compartment ID
│     ├─ reversible
│     ├─ reaction notes and description
│     ├─ list of reactants
│     ├─ list of products
│     └─ list of modifiers
│        ├─ ID
│        └─ type
├─ species database
│  └─ for every species ID
│     └─ species name
├─ name database
│  └─ for every species name
│     └─ species ID
├─ Ensembl database
│  └─ for every Ensembl ID
│     └─ gene name
└─ UniProt database
```
[cont.]

```
│
├─ for every UniProt ID
│  └─ protein name
├─ChEBI database
│  ├─ for every metabolite name
│  │  └─ ChEBI ID
├─ChEBI synonym database
│  ├─ for every ChEBI ID
│  │  └─ list of all associated metabolite names
├─UniProt metabolite database
│  ├─ for every UniProt metabolite name
│  │  └─ ChEBI ID
├─complex database
│  ├─ for every complex ID
│  │  ├─ complex ID
│  │  ├─ complex name
│  │  ├─ compartment ID
│  │  ├─ participating complex
│  │  ├─ pathway
│  │  ├─ top level pathway
│  │  ├─ list of ChEBI & UniProt & Ensembl IDs
│  │  ├─ list of UniProt participant IDs
│  │  ├─ list of Ensembl participant IDs
│  │  ├─ list of mirbase participant IDs
│  │  └─ list of NCBI participant IDs
├─compartment database
│  └─ for every compartment ID
│     └─ compartment name
```

Algorithm 2. `template.mvrs` graph structure.

```
│
├─ nodes
│  └─ for every reaction & species
│     ├─ map id
│     ├─ name
│     ├─ reversible
│     ├─ notes
│     ├─ type
│     ├─ sub-type
│     ├─ compartment
│     └─ compartment display name
└─ edges
   └─ for every edge tuple
      ├─ type
      └─ sub-type
```

Algorithm 3. `nbdb` reaction neighbors determination.

Require: $G = (V, E)$
Initialize: D ▷ D is a neighbors database

for $(e_{i,j}) \in E$ **do** ▷ E is a collection of directed edges in the graph (G)
  Update $D[e_i]$ with e_j
  Update $D[e_j]$ with e_i
  if $V[e_i]$ is `type:reaction` ▷ V is a collection of vertices in the graph (G) with node attributes
    for $x \in D[e_j]$ **do**
      if $V[D[e_j]_x]$ is `type:reaction`
        Update $D[e_i]$ with $D[e_j]_x$
      end if
    end for
  end if
  if $V[e_j]$ is `type:reaction`
    for $x \in D[e_i]$ **do**
      if $V[D[e_i]_x]$ is `type:reaction`
        Update $D[e_j]$ with $D[e_i]_x$
      end if
    end for
  end if
end for

Return: D

Collapsing reactions with missing expression or abundance values in user data

Additional parameters for the reaction-collapse are as follows:

1. If a reaction has at least one known or inferred value for inputs (substrates) and one known or inferred value for outputs (products), the reaction will be left as is. During the entire reaction collapse step, known catalysts can be included when assessing whether a reaction has measured output values (increased catalyst should lead to more output in most cases), and inhibitors can be included when assessing whether the reaction has measured input values (increased inhibitor should lead to an accumulation of input in most cases). Catalysts and inhibitors are not included when determining reaction neighbors, as described below.
2. If a reaction has at least one known input, the input is left as is, and each reaction that shares the same inputs with the first reaction's outputs is determined whether it has a measured output. If the neighbor reaction does not contain a known output value, the reaction is left as is. If the neighboring reaction does contain a measured output, the first reaction's inputs and the neighboring reaction's outputs are collapsed to form a single, pseudo-reaction between the two. If the reaction has at least one known output, the inverse is performed where neighbors with components identical to the reaction's inputs are assessed for whether a collapsed reaction can be created.
3. If a reaction has no measured values, it is determined if the neighboring reactions on both sides (one sharing the reaction's inputs and other sharing the reaction's outputs) have measured values. If both neighbors contain a measured value, a collapsed pseudo-reaction is created, summarizing all three reactions.
4. All other reactions are maintained in the network.

For collapsed reactions, appropriate notes are included to describe the collapse. During visualization, these collapsed reactions are marked by black dashed edges and dashed node borders. A visual summary of the reaction collapse procedure as outlined in Algorithm 4 can be found in Extended Data Fig. 2 and Extended Data Fig. 3.

Algorithm 4. Reaction collapse protocol.

Require: $G = (V, E); R; N$ ▷ R is a reaction database; N is a reaction neighbors database

Optional: $T_d = 50; T_c = 0.3$ ▷ T_d is the maximum node degree to consider when collapsing; T_c is the percentage of matching nodes between reactions to collapse
Initialize: U ▷ U is an updated reaction database

for $r \in R$ **do**
  if $\mathbb{R} \in r_i$ **and** $\mathbb{R} \in r_o$ ▷ No modification when both sides of reaction contain measured components
    Update U with r ▷ i is the set of reaction inputs (reactants) and o is the set of reaction outputs (products)
  end if
  if $\mathbb{R} \notin r_i$ **and** $\mathbb{R} \notin r_o$ ▷ Search for reactions where both inputs/outputs are not measured
    for $n_1 \in N$ **where** $n_{1_i} = r_i$ **or** $n_{1_o} = r_i$ **do**

for $n_2 \in N$ **where** $n_{2_i} = r_o$ **or** $n_{2_o} = r_o$ **do**
if $\mathbb{R} \in n_{1_i}$ **or** $\mathbb{R} \in n_{1_o}$ **and** $\mathbb{R} \in n_{2_i}$ **or** $\mathbb{R} \in n_{1_o}$
Update U with p_{n_1,n_2} ▷ p_{n_1,n_2} is the pseudo-reaction created from the measured inputs/outputs of n_1 and n_2
for $(i,o) \in p_{n_1,n_2}$ **do**
Update E with (i, p_{n_1,n_2}) or (p_{n_1,n_2}, o)
end for
end if
end for
end for
end if
if $\mathbb{R} \notin r_i$ ▷ Search for reactions where both inputs are not measured
for $n \in N$ **where** $n_i = r_i$ **or** $n_o = r_i$ **do**
if $\mathbb{R} \in n_i$ **or** $\mathbb{R} \in n_o$
Update U with $p_{r,n}$ ▷ $p_{r,n}$ is the pseudo-reaction created from the measured inputs/outputs of r and n
for $(i,o) \in p_{r,n}$ **do**
Update E with $(i, p_{r,n})$ or $(p_{r,n}, o)$
end for
end if
end for
end if
if $\mathbb{R} \notin r_o$ ▷ Search for reactions where both outputs are not measured
for $n \in N$ **where** $n_i = r_o$ **or** $n_o = r_o$ **do**
if $\mathbb{R} \in n_i$ **or** $\mathbb{R} \in n_o$
Update U with $p_{r,n}$
for $(i,o) \in p_{r,n}$ **do**
Update E with $(i, p_{r,n})$ or $(p_{r,n}, o)$
end for
end if
end for
end if
end for

Return: U

Regulatory pattern searches and sorting

Metaboverse provides a variety of different regulatory patterns for the user to explore. To identify a reaction-pattern is to compare some value that is computed from a reaction with a user-specified threshold (T). Reaction patterns available at the time of publication include Equations 1-11 (Supplementary Note 3). Reaction patterns values are calculated using the reactant (r), product (p), and/or modifier (m) sets for each reaction.

The identified reaction-patterns will be listed in a stamp view. Each stamp represents a reaction, with a glyph of the reaction, or the name of the pathway on it. In this stamp view, the identified patterns can be sorted according to three criteria: the number of pathways containing the reaction (not applicable for pathway pattern identification), the magnitude of the change of the computed value, and the statistical significance. When sorting by the number of pathways or the magnitude of the change, the identified reactions are arranged in order from the largest to the smallest. When sorting by the statistical significance, reactions with statistical significance on both the input side (substrates) and the output side (products) are listed first by the product of their maximum statistics, followed by the reactions with statistical significance on one of the two sides, and finally the reactions with no statistical significance on both sides. Within each tier, the reactions are sorted from lowest to highest p-values. For all values or statistics used in sorting, only those that determined the reaction-pattern are used. When sorting by FDR (false discovery rate), Metaboverse uses Equation 13 (Supplementary Note 3) to aggregate the p-values of reaction components that were relevant to the reaction pattern. Doing so also roughly leads to a generalized version of a false discovery rate procedure [24, 25].

When a reaction is selected from the stamp view, all the pathways containing the corresponding reactions will be listed below the stamp. Clicking on a pathway ID will draw the selected pathway in which the reaction-pattern was found, with all other reaction-patterns within this pathway also highlighted. For time-course and multi-condition datasets, the selected reaction-pattern's total behavior is displayed below these windows as line-plots showing the reaction components' behavior across all time-points or conditions.

Supplementary Note 3: Equations

Pattern search algorithms

A generalized expression of the pattern search algorithm:

$$\Big|\frac{1}{a}\sum_{i=1}^{a} p_i - \frac{1}{b}\sum_{j=1}^{b} r_j\Big| \geq T; \;\; \max_{|k| \in m} \geq T \tag{1}$$

Comparison of substrate/product averages (`Average`):

$$\Big|\frac{1}{a}\sum_{i=1}^{a} p_i - \frac{1}{b}\sum_{j=1}^{b} r_j\Big| \geq T \tag{2}$$

Sustained regulation (`Sustained`):

$$\Big|\frac{1}{a}\sum_{i=1}^{a} p_i\Big| \geq T \text{ and } \Big|\frac{1}{b}\sum_{j=1}^{b} r_j\Big| \geq T \text{ and } r \neq p \tag{3}$$

Modifier-driven regulation (`ModReg`):

$$\Big|\frac{1}{a}\sum_{i=1}^{a} p_i - \frac{1}{b}\sum_{j=1}^{b} r_j\Big| \geq T \text{ and } \max_{|k| \in m} \geq T \tag{4}$$

Transport regulation (`TransReg`):

$$\Big|\frac{1}{a}\sum_{i=1}^{a} p_i\Big| \geq T \text{ and } \Big|\frac{1}{b}\sum_{j=1}^{b} r_j\Big| \geq T \text{ and } r = p \tag{5}$$

Multi-step modifier regulation (`Enzyme`):

$$\begin{aligned} &\max_{|k| \in m} \geq T \text{ and } \max_{|l| \in m_N} \geq T \\ &\text{or} \\ &\big|(\mathtt{sign}(k) \cdot \max_{|k| \in m}) - (\mathtt{sign}(l) \cdot \max_{|l| \in m_N})\big| \geq T \end{aligned} \tag{6}$$

Multi-step metabolite regulation (`Metabolite`):

$$\max_{|i| \in p} \geq T \text{ and } \max_{|j| \in r} \geq T \text{ and } \max_{|w| \in p_N} \geq T \text{ or } \max_{|v| \in r_N} \geq T \tag{7}$$

Comparison of substrate/product maximums (`MaxMax`):

$$\max_{i \in p} - \max_{j \in r} \geq T \tag{8}$$

Comparison of substrate/product minimums (`MinMin`):

$$\min_{i \in p} - \min_{j \in r} \geq T \tag{9}$$

Comparison of substrate maximum and product minimum (`MaxMin`):

$$\min_{i \in p} - \max_{j \in r} \geq T \tag{10}$$

Comparison of substrate minimum and product maxiumum (`MinMax`):

$$\max_{i \in p} - \min_{j \in r} \geq T \tag{11}$$

where r denotes a set of reaction reactants, p denotes a set of reaction products, m denotes a set of reaction modifiers, which can each individually be considered within the reaction pattern, T denotes a user-specified threshold, and N indicates the components of a neighboring reaction. $\mathtt{sign}(k)$ or $\mathtt{sign}(l)$ indicates the sign of the maximum value of the associated set.

When a reaction is selected from the stamp view, all the pathways containing the corresponding reactions will be listed below the stamp. Clicking on a pathway ID will draw the selected pathway in which the reaction-pattern was found, with all other reaction-patterns within this pathway also highlighted. For time-course and multi-condition datasets, the selected reaction-pattern's total behavior is displayed below these windows as line-plots showing the reaction components' behavior across all time-points or conditions.

Reaction complex median

The median (Y) of all measured reaction component values X (metabolites, proteins, etc.) are calculated in order to weight the inferred value towards values of more frequent magnitude.

$$Y = \text{median}(X), \text{ where } X = \{x_1, x_2, ..., x_n\} \tag{12}$$

Reaction complex aggregated p-value

An aggregated p-value p_{agg} is inferred by multiplying the geometric mean of the p-values P by e, as in [24, 25]. This method was chosen as it: 1) implies dependence between p-values, as can be expected between the regulation of components of a protein complex, 2) weights the resulting p-value towards significance and prevents penalizing the complex's inferred p-value for one component with a poor p-value, and 3) ensures the resulting p-value is not lower than the minimum actual p-value from the set. Nodes for which values were inferred will be marked by a dashed border during visualization to clearly show which values are known and which were inferred. Statistical values are derived from the highest value of the components.

$$p_{agg} = (e * \sqrt[k]{P}), \text{ where } P = \{p_1, p_2, ..., p_n\} \text{ and } p_{agg} \in [0, 1] \tag{13}$$

References

1. B. Misra, C. Langefeld, M. Olivier, L. Cox. Integrated omics: tools, advances and future approaches. *J Mol Endocrinol* **62** (2018). https://doi.org/10.1530/JME-18-0055.
2. B. Misra. New software tools, databases, and resources in metabolomics: updates from 2020. *Metabolomics* **17** (2021). https://doi.org/10.1007/s11306-021-01796-1.
3. J. Xia, N. Psychogios, N. Young, D. Wishart. MetaboAnalyst: a web server for metabolomic data analysis and interpretation. *Nucleic Acids Res* **37** (2009). https://doi.org/10.1093/nar/gkp356.
4. J. Chong, *et al.*. MetaboAnalyst 4.0: towards more transparent and integrative metabolomics analysis. *Nucleic Acids Res* **46** (2018). https://doi.org/10.1093/nar/gky310.
5. Y. Zhou, *et al.*. Metascape provides a biologist-oriented resource for the analysis of systems-level datasets. *Nat Commun* **10** (2019). https://doi.org/10.1038/s41467-019-09234-6.
6. C. Wieder, C. Frainay, N. Poupin, et al. Pathway analysis in metabolomics: Recommendations for the use of over-representation analysis. *PLoS Comput Biol* **17** (2021). https://doi.org/10.1371/journal.pcbi.1009105.
7. P. Shannon, *et al.*. Cytoscape: A Software Environment for Integrated Models of Biomolecular Interaction Networks. *Genome Res* **13** (2003). https://doi.org/10.1101/gr.1239303.
8. S. Basu, *et al.*. Sparse network modeling and Metscape-based visualization methods for the analysis of large-scale metabolomics data. *Bioinformatics* **33** (2017). https://doi.org/10.1093/bioinformatics/btx012.
9. A. Karnovsky, *et al.*. Metscape 2 bioinformatics tool for the analysis and visualization of metabolomics and gene expression data. *Bioinformatics* **28** (2012). https://doi.org/10.1093/bioinformatics/btr661.
10. J. Gao, *et al.*. Metscape: a Cytoscape plug-in for visualizing and interpreting metabolomic data in the context of human metabolic networks. *Bioinformatics* **26** (2010). https://doi.org/10.1093/bioinformatics/btq048.
11. L. Cottret, *et al.*. MetExplore: a web server to link metabolomic experiments and genome-scale metabolic networks. *Nucleic Acids Res* **38** (2010). https://doi.org/10.1093/nar/gkq312.
12. L. Cottret, *et al.*. MetExplore: collaborative edition and exploration of metabolic networks. *Nucleic Acids Res* **46** (2018). https://doi.org/10.1093/nar/gky301.
13. M. Chazalviel, *et al.*. MetExploreViz: web component for interactive metabolic network visualization. *Bioinformatics* **34** (2018). https://doi.org/10.1093/bioinformatics/btx588.
14. G. Joshi-Tope, *et al.*. Reactome: a knowledgebase of biological pathways. *Nucleic Acids Res* **33** (2005). https://doi.org/10.1093/nar/gki072.
15. B. Jassal, *et al.*. The reactome pathway knowledgebase. *Nucleic Acids Res* **48** (2020). https://doi.org/10.1093/nar/gkz1031.
16. A. Fabregat, *et al.*. The Reactome Pathway Knowledgebase. *Nucleic Acids Res* **46** (2018). https://doi.org/10.1093/nar/gkx1132.
17. A. Wagner, *et al.*. Metabolic modeling of single Th17 cells reveals regulators of autoimmunity. *Cell* **184** (2021). https://doi.org/10.1016/j.cell.2021.05.045.
18. G. Chechik, *et al.*. Activity motifs reveal principles of timing in transcriptional control of the yeast metabolic network. *Nat Biotechnol* **26** (2008). https://doi.org/10.1038/nbt.1499.
19. T. Çakir, *et al.*. Integration of metabolome data with metabolic networks reveals reporter reactions. *Mol Syst Biol.* **2** (2006). https://doi.org/10.1038/msb4100085.
20. T. Çakir. Reporter pathway analysis from transcriptome data: Metabolite-centric versus Reaction-centric approach. *Sci Rep* **5** (2015). https://doi.org/10.1038/srep14563.
21. A. Kuehne, U. Mayr, D. Sévin, M. Claassen, N. Zamboni. Metabolic network segmentation: A probabilistic graphical modeling approach to identify the sites and sequential order of metabolic regulation from non-targeted metabolomics data. *PLoS Comput Biol.* **13** (2017). https://doi.org/10.1371/journal.pcbi.1005577.
22. A. Hagberg, D. Schult, P. Swart. Exploring Network Structure, Dynamics, and Function using NetworkX. *Proceedings of the 7th Python in Science conference (SciPy 2008)* (2008). http://conference.scipy.org/proceedings/SciPy2008/paper_2/full_text.pdf.
23. T. Waller, J. Berg, A. Lex, B. Chapman, J. Rutter. Compartment and hub definitions tune metabolic networks for metabolomic interpretations. *Gigascience* **9** (2020). https://doi.org/10.1093/gigascience/giz137.

24. L. Mattner. Combining individually valid and arbitrarily dependent P-variables. *Tenth German Probability and Statistics Days, Mainz, Germany* (2012). .

25. V. Vovk, R. Wang. Combining p-values via averaging. *Biometrika* **107** (2020). `https://doi.org/10.1093/biomet/asaa027`.
