# Supplementary material for: Metaboverse enables automated discovery and visualization of diverse metabolic regulatory patterns
Source: Nat Cell Biol. 2023 Apr 3;25(4):616–25. doi: 10.1038/s41556-023-01117-9 (PMC10104781; doi:10.1038/s41556-023-01117-9)
Supplement: Supplementary file 2 — Reporting Summary [file 41556_2023_1117_MOESM2_ESM.pdf]

nature portfolio

Corresponding author(s): Jordan A. Berg; Jared Rutter

Last updated by author(s): Feb 12, 2023

Reporting Summary

Nature Portfolio wishes to improve the reproducibility of the work that we publish. This form provides structure for consistency and transparency in reporting. For further information on Nature Portfolio policies, see our Editorial Policies and the Editorial Policy Checklist.

Statistics

For all statistical analyses, confirm that the following items are present in the figure legend, table legend, main text, or Methods section.

n/a	Confirmed	
☐	☒	The exact sample size (*n*) for each experimental group/condition, given as a discrete number and unit of measurement
☐	☒	A statement on whether measurements were taken from distinct samples or whether the same sample was measured repeatedly
☐	☒	The statistical test(s) used AND whether they are one- or two-sided<br>*Only common tests should be described solely by name; describe more complex techniques in the Methods section.*
☐	☒	A description of all covariates tested
☐	☒	A description of any assumptions or corrections, such as tests of normality and adjustment for multiple comparisons
☐	☒	A full description of the statistical parameters including central tendency (e.g. means) or other basic estimates (e.g. regression coefficient) AND variation (e.g. standard deviation) or associated estimates of uncertainty (e.g. confidence intervals)
☐	☒	For null hypothesis testing, the test statistic (e.g. *F*, *t*, *r*) with confidence intervals, effect sizes, degrees of freedom and *P* value noted<br>*Give P values as exact values whenever suitable.*
☒	☐	For Bayesian analysis, information on the choice of priors and Markov chain Monte Carlo settings
☒	☐	For hierarchical and complex designs, identification of the appropriate level for tests and full reporting of outcomes
☐	☒	Estimates of effect sizes (e.g. Cohen's *d*, Pearson's *r*), indicating how they were calculated

Our web collection on statistics for biologists contains articles on many of the points above.

Software and code

Policy information about availability of computer code

Data collection	Metaboverse network files were prepared using Metaboverse v0.9.0 and v0.10.0, as specified in the manuscript.<br>No other data collection or preparation software was used.
Data analysis	Metaboverse analysis was performed using Metaboverse v0.9.0 and v0.10.0, as specified in the manuscript.<br><br>Additional source code generated for the analyses within this manuscript are available at https://github.com/Metaboverse/Metaboverse-manuscript/. Dependency version numbers are printed within code notebooks.<br><br>List of software dependencies and versions (as available):<br>Metaboverse (v0.9.0, v0.10.0)<br>R (v4.0.3)<br>survival (v3.2-11)<br>survminer (v0.4.9)<br>ggpubr (v0.4.0)<br>XPRESSpipe (v0.6.0)<br>DESeq2 (v1.22.1)<br>dupRadar (v1.14.0)<br>fastp (v0.20.0)<br>star (v2.7.3a)<br>bioconductor-rsamtools (v1.34.0)<br>samtools (v1.10)

bedtools (v2.29.2)
fastqc (v0.11.9)
htseq (v0.11.3)
matplotlib (v3.1.2, v3.4.2)
matplotlib-base (v3.1.1)
pandas (v1.0.2)
numpy (v1.17.4)
numpy-base (v1.17.4)
numpydoc (v0.9.2)
scipy (v1.4.1)
scikit-learn (v0.22.1)
multiqc (v1.8)
xpressplot (v0.2.4)
seaborn (v0.10.0, v0.11.0)
SpQN (v1.0.0)
Gene Ontology (GO) Resource (Release 2021-05-01)
PANTHER Overrepresentation Test (v16; Release 20210224)
El-MAVEN (v0.12.0)
MassHunter
Adobe Illustrator
Microsoft Excel

For manuscripts utilizing custom algorithms or software that are central to the research but not yet described in published literature, software must be made available to editors and reviewers. We strongly encourage code deposition in a community repository (e.g. GitHub). See the Nature Portfolio guidelines for submitting code & software for further information.

Data

Policy information about availability of data

All manuscripts must include a data availability statement. This statement should provide the following information, where applicable:

- Accession codes, unique identifiers, or web links for publicly available datasets
- A description of any restrictions on data availability
- For clinical datasets or third party data, please ensure that the statement adheres to our policy

Gene expression counts for lung adenocarcinomas were obtained from the Human Protein Atlas project's TCGA FPKM gene expression data (https://www.proteinatlas.org/download/rna_cancer_sample.tsv.zip) and clinical patient data were obtained from TCGA (https://portal.gdc.cancer.gov/projects/TCGA-LUAD). Single cell data were obtained from the Human Lung Cell Atlas project v1.0 (https://zenodo.org/record/6337966#.YkzVrOjMIQ-). DepMap data 21Q4 Public was used.

mct1Δ and accompanying wild-type transcriptomics time-course data are deposited at the GEO repository under identifier GSE151606. mct1Δ and wild-type proteomics data are deposited at the ProteomeXchange repository under identifier PXD035000. Metabolomics data
are deposited at the Metabolomics Workbench repository under project identifier PR000961, study identifier ST001401 and project identifier PR001422, study identifier ST002232. For gene co-expression analyses, all yeast samples available in refine.bio were accessed and downloaded on March 16, 2021.

Human research participants

Policy information about studies involving human research participants and Sex and Gender in Research.

Reporting on sex and gender	Not applicable
Population characteristics	Not applicable
Recruitment	Not applicable
Ethics oversight	Not applicable

Note that full information on the approval of the study protocol must also be provided in the manuscript.

Field-specific reporting

Please select the one below that is the best fit for your research. If you are not sure, read the appropriate sections before making your selection.

☒ Life sciences ☐ Behavioural & social sciences ☐ Ecological, evolutionary & environmental sciences

For a reference copy of the document with all sections, see nature.com/documents/nr-reporting-summary-flat.pdf

Life sciences study design

All studies must disclose on these points even when the disclosure is negative.

Sample size	For yeast experiments, samples were prepared with separate and fresh preparations with 3-6 biological replicates in each experimental or control group, as detailed in the Methods section and elsewhere as appropriate within the manuscript. In the case of the refine.bio yeast cohort, the entire wild-type sample cohort was used as specified in the manuscript text. For the public human lung adenocarinoma datasets, the Wikoff 2015 study contained 39 tumor tissue samples and 39 paired normal tissue samples; and TCGA data contained 487 gene expression samples total that were relevant to this study.<br><br>No statistical method was used to predetermine sample size. Sample sizes for high-throughput data generated for this study were chosen based on first-principles understanding of the number of samples needed to generate expected statistical distributions based on the data type. Statistical values were then adjusted for false positives following the convention for the respective data type. Other data were previously generated for other studies.
Data exclusions	For survival analysis, TCGA data were right censored and then removed if no days to death or censored days to death were available. Metabolomics samples that did not pass basic QC (n=1) were excluded from further analysis. No additional data were excluded.
Replication	All biological assays were repeated at least 3 times. All replication attempts were successful. Verification of plasmid construct expression by western blot was performed once as a simple validation that the construct was being over-expressed.
Randomization	Samples were randomized during sample preparation, but not during sample harvest for the yeast RNA sequencing and yeast metabolomics data, or were previously collected (human TCGA data, human metabolomics data, and yeast proteomics data), or otherwise not amenable to randomization (yeast growth spot tests, etc.). Yeast sample ordering and handling would have otherwise been randomized during sample processing.
Blinding	Data were either previously collected (human TCGA data, human metabolomics data, and yeast proteomics data), blinded during sample preparation but not sample harvest (yeast RNA sequencing, yeast metabolomics), or were otherwise not amenable to blinding (yeast growth spot tests, etc.). Yeast samples were additionally difficult to blind during growth and harvest as exact growth rates need to be measured throughout, and often correlate with genetic background.

Reporting for specific materials, systems and methods

We require information from authors about some types of materials, experimental systems and methods used in many studies. Here, indicate whether each material, system or method listed is relevant to your study. If you are not sure if a list item applies to your research, read the appropriate section before selecting a response.

Materials & experimental systems

n/a	Involved in the study	
☐	☒	Antibodies
☒	☐	Eukaryotic cell lines
☒	☐	Palaeontology and archaeology
☒	☐	Animals and other organisms
☒	☐	Clinical data
☒	☐	Dual use research of concern

Methods

n/a	Involved in the study	
☒	☐	ChIP-seq
☒	☐	Flow cytometry
☒	☐	MRI-based neuroimaging

Antibodies

Antibodies used	α-GFP (Cell Signaling Technology #2956) and α-Pgk1 (Abcam #ab113687)
Validation	α-GFP (Cell Signaling Technology #2956): Rabbit. RRID: AB 1196615. 1/2000 dilution dilution used in this study. Applications: WB & IHC. Reactivity: Saccharomyces cerevisiae. Datasheet available at https://www.cellsignal.com/datasheet.jsp?productId=2956&images=1<br>α-Pgk1 (Abcam #ab113687): Mouse Primary Monoclonal (22C5D8), Unconjugated, Unmodified. RRID: AB 10861977. 1/3000 dilution used in this study. Applications: WB & ICC-IF. Reactivity: Saccharomyces cerevisiae. No validations available, but cited many times in literature (https://www.citeab.com/antibodies/746015-ab113687-anti-pgk1-antibody-22c5d8).
