# Supplementary material for: Metaboverse enables automated discovery and visualization of diverse metabolic regulatory patterns
Source: Nat Cell Biol. 2023 Apr 3;25(4):616–25. doi: 10.1038/s41556-023-01117-9 (PMC10104781; doi:10.1038/s41556-023-01117-9)
Supplement: Source Data Fig. 5 — Source numerical data. [file 41556_2023_1117_MOESM7_ESM.zip › fig_5_source/mns/outputs/doc/Metabolic network segmentation toolbox v1.0 - documentation.pdf]

Metabolic network segmentation toolbox v1.0

Documentation

Andreas Kühne, 26th July 2016

For questions, comments or suggestions please contact kuehne@imsb.biol.ethz.ch or zamboni@imsb.biol.ethz.ch

Contents

Contents 2

Overview of the toolbox 4

Installation of the toolbox 4

MNS for univariate data: Identification of sites of metabolic regulation 4

Metabolic model 5

MNS-data structure 5

Initialization of model parameterization 6

Run the MNS inference to identify sites of regulation 8

Single model parameterization 9

Multiple model parameterization 9

Functions to analyze the output of the MNS inference for univariate data 9

Generate overview of reactions sorted by fracture stability. 9

Find gene rank 10

Export segmentation results to Cytoscape 10

Show metabolite changes around a specific reaction in a Matlab biograph 11

Export metabolite changes around a specific reaction to cytoscape 11

Example 1: Identification of regulatory sites in fibroblasts with transketolase knockdown using MNS for univariate data with a single parameterization 12

Example 2: Identification of regulatory sites in E. coli with purM knockout using MNS for univariate data with a single parameterization 15

MNS for sequential data to identify sites and sequential order of metabolic regulation 17

MNS-data structure 17

Initialization of model parameterization 17

Run the MNS inference to identify sites and sequential order of regulation 17

Functions to analyze the output of the MNS inference for sequential data 18

Plot fracture frequency and sum of observation potential 18

Plot score distributions for increasing weights ws and wn 19

Plot score distribution and segmentation results for given weights ws and wn 20

Plot module labels and fractures of MNS inference results for range of weights ws and wn 20

Plot sequential metabolite data with module label overlay 21

Example 3: Identification of regulatory sites in fibroblasts with transketolase knockdown using MNS for univariate data with a single parameterization 21

Copyright and License 30

Appendix 1 31

Observation potential function type 31
Neighborhood potential function type 32

Overview of the toolbox

The Metabolic Network Segmentation (MNS) toolbox contains algorithms employing Markov random fields to identify sites and sequential order of metabolic regulations from large-scale metabolomics datasets and genome-scale metabolic network reconstructions. The toolbox is implemented in Matlab and calls specific C++ functions of the OpenGM toolbox (http://hciweb2.iwr.uni-heidelberg.de/opengm/). The toolbox can be run on Windows and Mac operating systems.

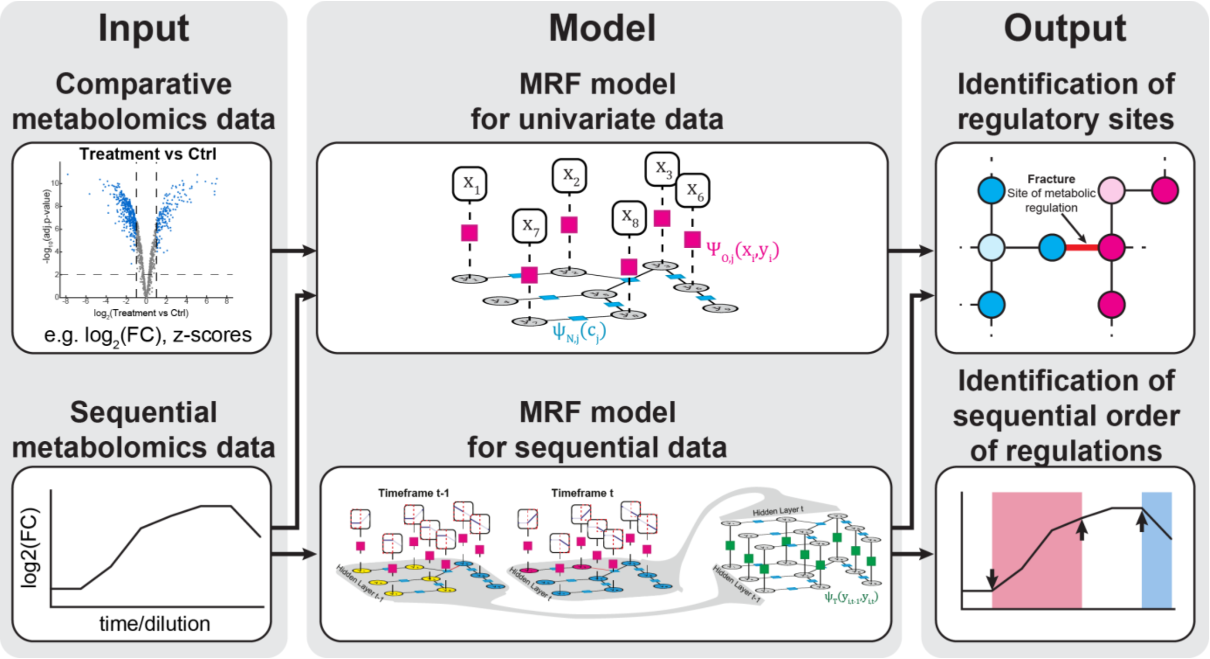

Installation of the toolbox

Copy/unpack the MNS toolbox on your hard drive. Start Matlab, go to the folder of the MNS toolbox and initialize the toolbox by executing

```
mns_initialize()
```

The MNS toolbox needs to be initialized always when Matlab is restarted.

MNS for univariate data: Identification of sites of metabolic regulation

The MNS for univariate data takes as input comparative metabolomics data, such as log2(fold-change) comparing metabolite measurements from treated and control samples or z-scores, as well as metabolic network reconstruction to identify sites of metabolic regulations. The method needs as input a metabolic model and a MNS-data structure. Before the inference, the model parameterization needs to be ina

Metabolic model

We use KEGG main reactant pair models for the MNS toolbox. The toolbox comes with models for several organisms (e.g. E. coli, Homo sapiens). However, in general the methods can be run with any metabolic model. The model structure in Matlab needs to have the following fields:

Field	Variable Type	Description
iaMat	MxM double	interaction matrix of metabolites (0 = no interaction, 1 = reactant-pair), M = total number of metabolites
mat	RxM double	interaction matrix of reactions and metabolites (0 = not involved, 1 = metabolite is involved in reaction), R = total number or reactions
metaboliteId	Mx1 cell	Metabolite Identifier
metaboliteName	Mx1 cell	Metabolite Name
rpId	1xR cell	Reaction Identifier
ECtoRP	ExR double	connection between EC numbers and reactions (0 = no connection, 1 = reaction belongs to EC number), E = total number of EC numbers
GeneToEC	GxE double	connection between Genes and EC numbers (0 = no connection, 1 = Gene encodes enzyme with functionality of EC number), G = total number of genes
EC	Ex1 cell	EC numbers
Gene	Gx1 cell	Gene identifiers
GeneSymbol	Gx1 cell	Gene symbol

MNS-data structure

The MNS-data structure needs to have the following fields:

Field	Variable Type	Description
dataType	string	Tag describing the type of the input data. Options<br>*MetIdList* - metabolite annotation consist of m x 1 vector of metabolite ids<br>*fiaExp* - (internal use only) for fiaMiner annotation version 2.0<br>*fiaExp v3.0* - (internal use only) for fiaMiner annotation version 3.0
data	m x 1 double or m x s double	Data matrix of comparative metabolomics data. The MNS approach for univariate data requires a m x 1 double vector; m = number of metabolites
annotation	m x 1 cell of string or struct	Metabolite annotation. For option MetIdList annotation is a m x 1 cell vector of metabolite ids. Internal: For option fiaExp annotation represents the annotation field of die fiaExp structure, for option fiaExp v3.0 annotation has the fields annotation and anndata that are the ones from the fiaExp

For the univariate MNS method any comparative data type, such as such as log2(fold-change) comparing metabolite measurements from treated and control samples or z-scores, can be used.

Initialization of model parameterization

The model parameterization initMNS can be initialized with

```
initMNS = mns_generateInitMNS(ParameterName, Value)
```

The initMNS structure has the following fields:

Field	Variable Type	Default Value	Description
noOfclusters	double	3	Number of hidden state labels, i.e. number of module types
nL1steps	double	40	Maximal number of scanning steps (lambda1).
meanType	String	initLabels	Describes how the mean values for the hidden state dependent Gaussian functions are determined. Options:<br>• *initLabels* - mean determined by groups defined by the initial labels e.g. through k-means<br>• *linear - std* - means linearly distributed between min(data)+std and max(data)-std<br>• *linear - quantile* - means linearly distributed between quantile(data, initMNS.mean) and quantile(data, 1-initMNS.mean)<br>• *fix* - means as defined in initMNS.mean
mean	double	[]	For meanType = 'fix', mean is a vector of size 1xnoOfclusters describing the mean values of the hidden state dependent gaussian functions. For meanType = 'linear – quantile' it is a 1x1 double value describing the quantile range.
stdType	String	fix	Describes how the standard deviation values for the hidden state dependent Gaussian functions are determined. Options:<br>• *'fix'* – data independent value as defined in stdVal<br>• *'one group'* – Standard deviation for all hidden states is similar and set to the average of the standard deviations of the metabolite groups determined by k-mean clustering.<br>• *'multiple group'* - Standard deviation for all hidden states is individual and set to the standard deviation of the metabolite groups determined by k-mean clustering.<br>• *'one group – factor'* – Similar to *one group* option but the standard deviation value gets multiplied by a factor defined in stdVal

Field	Variable Type	Default Value	Description
			• *'multiple group - factor'* – Similar to *multiple group* option, but the standard deviation value gets multiplied by a factor defined in stdVal<br>• *'one group - data - factor'* – Standard deviation for all hidden states is similar and set to the the standard deviations of the whole dataset. The value gets multiplied by a factor defined in n stdVal
stdVal	double	1	See stdType description for details
normObsProb	double	2	Type of the observation potential function. Options (See Appendix 1 for details):<br>• *1* – normalized Gaussian<br>• *2* – not normalized Gaussian<br>• *3* – normalized Gaussian and linkage dependent gaussian<br>• *4* – not normalized Gaussian and linkage dependent gaussian
neighProbFuncType	double	1	Type of the neighborhood potential function. Options:<br>• *0* – clique size normalized<br>• *1* – clique size normalized and normalized to 1<br>• *2* – not clique size normalized<br>• *3* – not clique size normalized and normalized to 1<br>See Appendix 1 for details
initLabelsType	String	ones	Determines how the initial hidden state/module labels are defined. Options:<br>• *'random'* – initial labels are randomly picked for each of a uniform distribution between 0 and noOfclusters-1<br>• *'zeros'* – all initial labels are set to 0<br>• *'ones'* – all initial labels are set to 1<br>• *'middle cluster'* – all initial labels are set to the to the cluster with the average mean metabolite change<br>• *'initLabels'* – all initial labels are set to the cluster affiliation as determined by k-means
initLabelsTypeApplyToAll	logical	1	Determines if the initial label types is applied to all (*1*) or only undetected metabolites (*0*)
input	struct	-	Structure with certain input parameter for the inference. Fields:<br>• *'inferenceParameter'* – number of neighborhood nodes to consider for LazyFlipper inference. Default: 2<br>• *'inferenceAlgorithm'* – solver type from openGM to optimize the Markov random

Field	Variable Type	Default Value	Description
			field model. Options: *'LazyFlipper'* (Default), *'LBP'* (Loopy Belief Propagation), *'ASTAR'*, *'ICM'* (Iterated conditional modes)<br>• *'dataFolder'* – path to folder to write and read the temporary data for MNS inference. If '' (empty) it uses the data folder of the MNS toolbox. This is essential for analysis using a cluster<br>• *'mnsExecFolder'* – path to folder containing the executable for MNS inference using the openGM toolbox. If '' (empty) it uses the openGM folder of the MNS toolbox. This is essential for analysis using a cluster.
determinePvalue	double	0	Determines if *p-values* are calculated using a permutation test:<br>• *'0'* – no p-value determination<br>• *'2'* – p-values are determined through random permutation of metabolite levels
permutations	double	1000	Number of permutations for p-value determination
parallel	struct	-	The p-value determined can be run on a cluster. The parallel structure has 5 fields in that need to be defined:<br>• *'useCluster'* – if true (1) use cluster, if false (0) not<br>• *'clusterName'* – name of the Matlab cluster<br>• *'cores'* – number of cores<br>• *'parallelFolder'* – path on which the cluster have read and write rights to perform inference. If the cluster runs locally this is not necessary<br>• *'parallelFolderCopy'* – path that gives access from local computer to cluster path defined in *parallelFolder*
verbose	Logical	0	If true (1) C++ MNS inference generates output on screen
verboseScan	double	2	Show screen output of the MNS: scanning procedure. Options<br>• *0* – no output<br>• *1* – summary output<br>• *2* – detailed output

Without input arguments initMNS is set to the default parameters. The parameters can be adapted directly using fieldname, value pairs in the mns_generateInitMNS function.

Run the MNS inference to identify sites of regulation

The inference can be run with a single or with multiple parameterizations.

Single model parameterization

The inference with a single parameterization can be run using

```
mnsScanResults = mns_scan2state(metabolic_model, MNS_dataStruct,
nameTag, l1Range, initMNS, plotResults)
```

Only the first two Input arguments (metabolic_model, MNS_dataStruct) are required and need to be structured as described before. If the other variables are not defined they are set to the default values. Description of other Input arguments:

Input argument	Default	Description
nameTag	'temp'	Name of the folders in which the temporary data is stored.
l1Range	0	Scanning range of the lambda 1 (neighborhood influence parameter), e.g [0:0.1:2]. If set to 0 (default), the range is determined automatically till nor more fractures are identified
plotResults	0	If 1 plots output results of the MNS scan

Multiple model parameterization

The inference with a multiple parameterization can be run using

```
mnsScanResults = mns_scan2state_multipleParameterizations(metabolic_model,
MNS_ataStruct, nameTag, l1Range, initMNS_1, initMNS_2, ... , initMNS_n)
```

The input arguments are similar to the ones for the single parameterizations. If the nametag and l1Range parameters are not defined (empty), they are set automatically to the default values. To run the MNS inference with n-different parameterizations, add the n-different initMNS structures as last input arguments.

Functions to analyze the output of the MNS inference for univariate data

Generate overview of regulated reactions sorted by fracture stability.

```
resultsTable = mns_scanResult2table(mnsScanResults, metabolic_model,
sortBy, modelType)
```

Only the first two Input arguments are required. Description of Input arguments

Input argument	Default	Required	Description
mnsScanResults	-	x	Name of the folders in which the temporary data is stored.
metabolic_model	-	x	Metabolic Model
sortBy	'rankMax'		Metric according to which the list is sorted. Options:<br>• *'rankMax'* – ranked according to max lambda1 at which a fracture exists

			• *'rankSum'* – ranked according to the total number of fractures<br>• *'rankproductMax'* – rank according to rankproduct of the ranking according to max lambda1. Only available for MNS inference with multiple parameterizations.<br>• *'rankproductSum'* – rank according to rankproduct of the ranking according to total number of fractures. Only available for MNS inference with multiple parameterizations.
modelType	'KEGGS'		Type of the metabolic model. So far only implemented for KEGG main reactant pair models (*'KEGGS'*)

The output table contains the following fields: Reaction rank, Reaction pair ID (RP ID), EC number, Gene symbol, Reaction, Max lambda1, p(Max lambda1), total no of fracture, p(total no of fractures).

Find gene rank

This function identifies the best rank of a certain gene and it's k-st neighbor. The output is the best rank according to max lambda1 (rankMax) and to total number of fractures (rankSum).

```
[rankSum, rankMax] = mns_scanFindGeneRank(mnsScanResults,metabolic_model,
geneSymbol,k)
```

Input argument	Default	Required	Description
mnsScanResults	-	x	Name of the folders in which the temporary data is stored.
metabolic_model	-	x	Metabolic Model
geneSymbol	-	x	Gene Symbol of the gene of interest
k	-	x	Analyze ranks till k-nearest neighbor reaction of gene of interest

Export segmentation results to Cytoscape

Export the segmentation results of the MNS inference for visualization in Cytoscape to excel worksheets (www.cytoscape.org).

```
mns_scan2state2cytoscape(metabolic_model, mnsScanResults, nameBase)
```

Input argument	Default	Required	Description
mnsScanResults	-	x	Name of the folders in which the temporary data is stored.
metabolic_model	-	x	Metabolic Model
nameBase	'temp_mns'		• Name base for filename of excel worksheets

Show metabolite changes around a specific reaction in a Matlab biograph

Show the inference results of the x-th ranked reaction with metabolite changes in the context of the metabolic network

```
mns_scanResults2biograph(metabolic_model, mnsScanResults, sortBy, idx,
nNeighborMetabolites)
```

**Input argument**	**Default**	**Required**	**Description**
mnsScanResults	-	x	Name of the folders in which the temporary data is stored.
metabolic_model	-	x	Metabolic Model
sortBy	'rankMax'		Metric according to which the list is sorted. Options:<br>• *'rankMax'* – ranked according to max lambda1 at which a fracture exists<br>• *'rankSum'* – ranked according to the total number of fractures
idx	1		Identifier of the x-ranked reaction to be visualized
nNeighborMetabolites	10		Show n neighbouring metabolites

Export metabolite changes around a specific reaction to cytoscape

Export the inference results of the x-th ranked reaction with metabolite changes to excel worksheets for visualization in Cytoscape. (www.cytoscape.org).

```
mns_scanResultHits2cytoscape(metabolic_model, mnsScanResults, nameBase,
sortBy, idx, nNeighborMetabolites)
```

**Input argument**	**Default**	**Required**	**Description**
mnsScanResults	-	x	Name of the folders in which the temporary data is stored.
metabolic_model	-	x	Metabolic Model
sortBy	'rankMax'		Metric according to which the list is sorted. Options:<br>• *'rankMax'* – ranked according to max lambda1 at which a fracture exists<br>• *'rankSum'* – ranked according to the total number of fractures
nameBase	'temp_mns'		Name base for filename of excel worksheets
idx	1		Identifier of the x-ranked reaction to be visualized
nNeighborMetabolites	10		Show n neighbouring metabolites

Example 1: Identification of regulatory sites in fibroblasts with transketolase knockdown using MNS for univariate data with a single parameterization

The analysis in this example is run on a reduced metabolic model of the pentose phosphate pathway. The metabolomics data is from a comparison between fibroblasts with a transketolase knockdown compared to wildtype fibroblasts. Both cell types were treated with hydrogen peroxide to activate flux through the pentose phosphate pathway (Data from Kuehne et al, Mol Cell. 2015 Aug 6;59(3):359-71). The example can be found in the example folder in 'mns_example_univariate_mns _fibroblasts_TK_knockdown_script.m'

```
%% load the data and model
load('mns_example_univariate_mns_fibroblasts_TK_knockdown - WS.mat')

%% initialize mns
%go to mns folder and exectute
mns_initialize


%% run MNS inference and calculate p-values
%
data = dataStructTK;
model = KEGG_HSA_MNS_redGlycPPP;
model.metaboliteName = metAbbreviations;

% initalize MNS parameterization
% change number of hidden states to 5
% calculate the p-value
initMNS = mns_generateInitMNS('noOfclusters', 5, 'determinePvalue', 2);

% run MNS inference
mnsScanResultsTKKD = mns_scan2state(model,data, 'TKKD', 0, initMNS, true);

% change colormap of module label distribution
cmap = [236 0 140; 46 49 146; 0 174 239; 0 166 81;255 242 0]/255;
colormap(cmap)
```

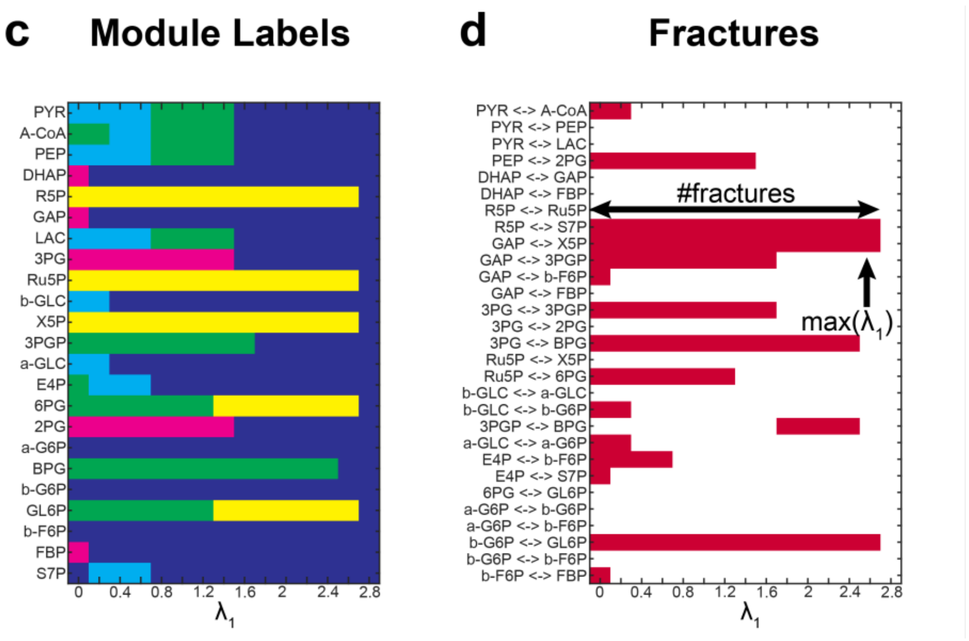

The outcome plot shows how the module label (left) and fracture (red, right) distribution changes with increasing neighborhood influence lambda1. Only the most stable reactions remain.

```
%% generate outable of the inference results sorted according rankSum
tkkd_results_table = mns_scanResult2table(mnsScanResultsTKKD, model,
'rankSum');
```

Rank	RP ID	EC	Gene Symbol	Reaction	max lambda1	p(max lambda1)	#fractures	p(#fractures)
2.5	RP01654	2.2.1.1	TKT; TKTL1; TKTL2	R5P <-> S7P	2.8	0.005	15	0.004
2.5	RP00080	2.2.1.1	TKT; TKTL1; TKTL2	GAP <-> X5P	2.8	0.005	15	0.004
2.5	RP01567	3.1.3.13	BPGM	3PG <-> BPG	2.8	0.005	15	0.004
2.5	RP02450	1.1.1.49	G6PD	b-G6P <-> GL6P	2.8	0.005	15	0.004
5.5	RP00395	1.2.1.12	GAPDH; GAPDHS	GAP <-> 3PGP	1.8	0.092	10	0.078
5.5	RP00113	2.7.1.106;	PGM2L1; PGK1; PGK2; ACYP1; ACYP2	3PG <-> 3PGP	1.8	0.092	10	0.078
7	RP01033	4.2.1.11	ENO1; ENO2; ENO3	PEP <-> 2PG	1.4	0.206	8	0.19
8	RP01572	1.1.1.44	PGD	Ru5P <-> 6PG	1.2	0.295	7	0.269
9	RP01676	5.4.2.4	BPGM	3PGP <-> BPG	2.8	0.005	5	0.518
10	RP01790	2.2.1.1	TKT; TKTL1; TKTL2	E4P <-> b-F6P	0.6	0.744	4	0.709

The results table shows that Transketolase is the top ranked gene.

```
%% find the rank of a certain gene: TKT (Transketolase)
[rankSum, rankMax] = mns_scanFindGeneRank(mnsScanResultsTKKD, model,'TKT',
1);
disp(['Best rank of TKT according to total number of fractures: '
num2str(rankSum(1))]);
```

Identify the best rank of a reaction catalyzed by the transketolase gene (TKT).

```
%% Show the metabolite changes around the first ranked reactions in a
biograph
mns_scanResults2biograph(model, mnsScanResultsTKKD, 'rankSum', 1, 6)
```

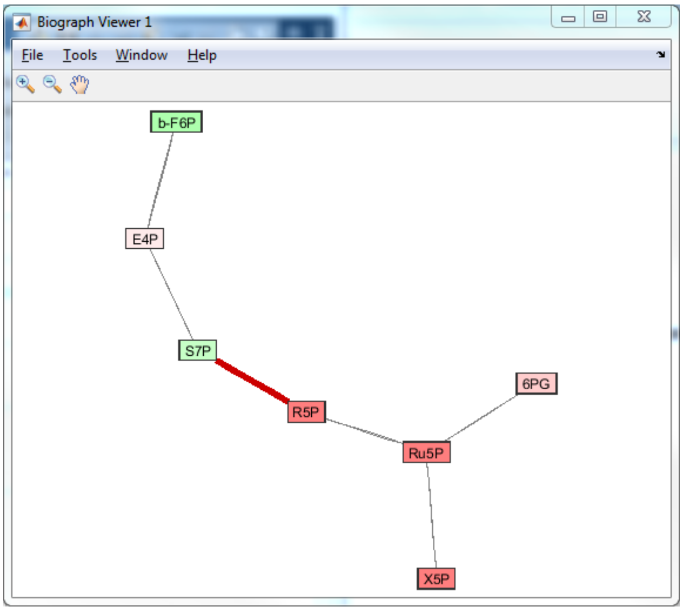

The graph shows the reaction pair between Ribose-5-P and Sedoheptulose 7-phosphate and the surrounding metabolite changes.

```
%% save the results in excel worksheets for import to cytoscape
% http://www.cytoscape.org/
model = KEGG_HSA_MNS_redGlycPPP;
model.metaboliteName = metAbbreviations;
mns_scan2state2cytoscape(model, mnsScanResultsTKKD, 'MNS Example - TKKD')
```

In cytoscape one can visualize the metabolic with the comparative metabolomics data

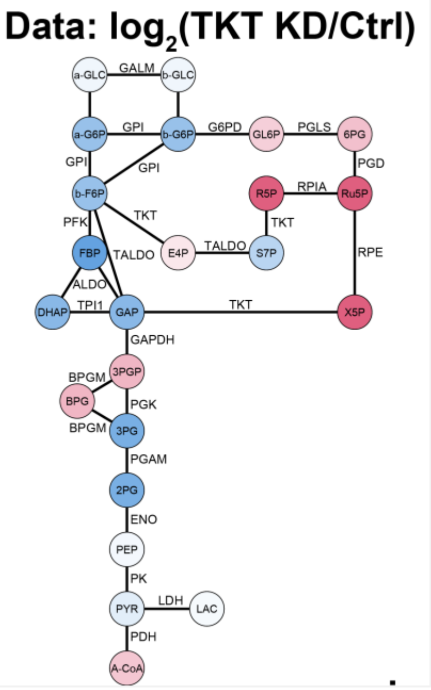

and the segmentation results and different strength of neighborhood influences, i.e. different values of lambda1

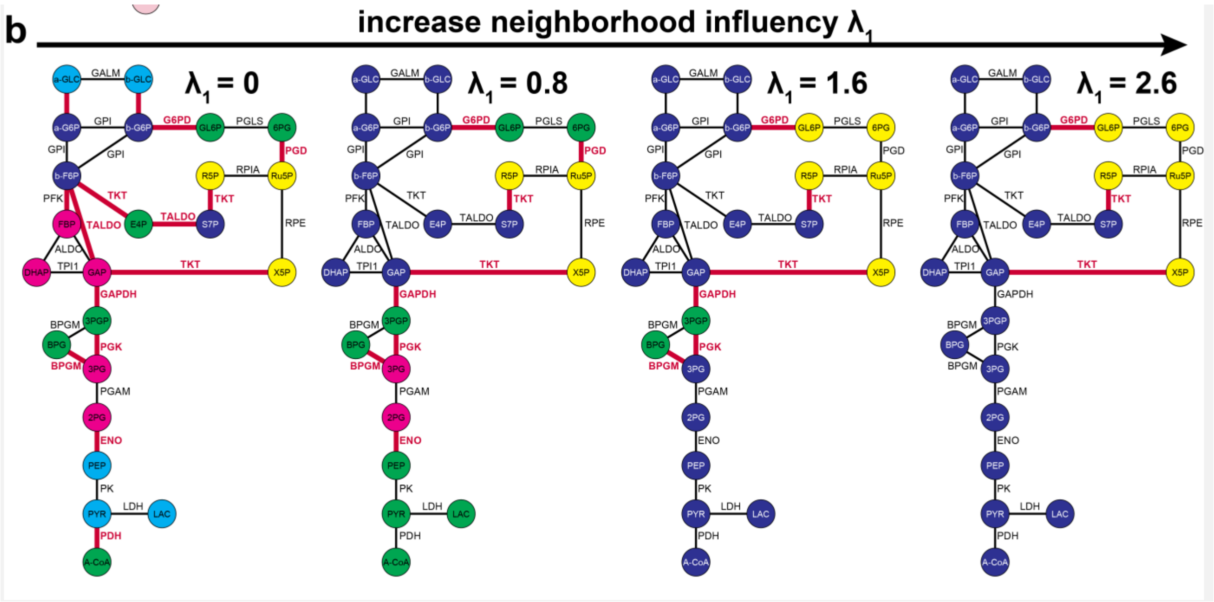

In this example it becomes obvious how the increasing neighborhood influence removes not relevant fractures and reveals the regulated reactions that are biologically relevant that are in this case the reactions of the perturbed transketolase enzyme.

Example 2: Identification of regulatory sites in E. coli with purM knockout using MNS for univariate data with a single parameterization

The analysis in this example is run on a full KEGG main reactant pair model for *E. coli* and with multiple parameterizations of the MNS model. The metabolomics data is from a comparison between E. coli with purM knockout and wild type *E. coli* (Data from Kuehne et al, unpublished). The example can be found in the example folder in ‘mns_example_univariate_mns_multiple _param_ecoli_purM_ko_script’.

```
%% initialize MNS
% go to mns folder and perform the execute command:
mns_initialize

%% load the data
% the datastructure contains log2(FC) data comparing E. coli + glucose with
% purM KO and wild type E. coli both cultured in M9 minimal medium
load('mns_example_univariate_mns_multiple_param_ecoli_purM_ko - WS')

%% load the model
% KEGG Ecoli
load KEGG_ECO_MNS
model = KEGG_ECO_MNS;

%% initialize different MNS parameterizations

% generate initMNS_3
% (parameter combination 3)
initMNS_2 = mns_generateInitMNS('verboseScan', 0, 'stdType', 'one group');

% generate initMNS_3
% (parameter combination 3)
```

```
initMNS_3 = mns_generateInitMNS('verboseScan', 0, 'stdType', 'one group - all data - factor'...
    ,'stdVal',1);

%% run MNS inference for multiple parameterizations
mnsScanResults_p2_3 =
mns_scan2state_multipleParameterizations(model,dataStruct_purM_KO,'purM_KO', 0, initMNS_2, initMNS_3);
```

This part of the code loads all the data generates two different MNS parameter initializations and runs the inference. The results can be analyzed using the following functions:

```
%% find gene rank of purM KO
[rankSum, rankMax] = mns_scanFindGeneRank(mnsScanResults_p2_3,model,
'purM', 1);
disp(['Best rank of purM KO according to rankproduct of total number of fractures: ' num2str(rankSum(1))]);

>> Best rank of purM KO according to rankproduct of total number of fractures: 2.5
```

Identify the best rank according to rankproduct of total number of fractures of a reaction catalyzed by the purM gene.

```
%% generate results table
resultsTable = mns_scanResult2table(mnsScanResults_p2_3,
model,'rankproductSum');
```

Rank	RP ID	EC	Gene Symbol	Reaction	rp(max lambda1)	p(rp(max lambda1))	rp(#fractures)	p(rp (#fractures))
1	RP01224	1.1.1.85	leuB	2-Oxobutanoate <-> D-erythro-3-Methylmalate	2.20E+01	2.74E-05	1.45E+01	1.60E-05
2.5	RP02160	3.1.3.6	cpdB	Cytidine <-> 3-CMP	99	0.000174502	5.08E+01	7.77E-05
2.5	RP03838	6.3.3.1	purM	Aminoimidazole ribotide <-> 2-(Formamido)-N1-(5-phosphoribosyl)acetamidine	99	0.000174502	5.08E+01	7.77E-05
4	RP01810	2.4.2.10	pyrE	Orotate <-> Orotidine 5-phosphate	99	0.000174502	87	0.000149451
5.5	RP03148	1.1.1.95	serA	2-Oxoglutarate <-> 2-Hydroxyglutarate	176	0.000345392	108.75	0.000195233
5.5	RP00423	2.1.3.2	pyrB	L-Aspartate <-> N-Carbamoyl-L-aspartate	99	0.000174502	108.75	0.000195233
7	RP15642	6.3.4.20	queC	7-Cyano-7-carbaguanine <-> 7-Carboxy-7-carbaguanine	198	0.000396671	130.5	0.00024254
8	RP15604	3.5.1.110	rutB	(Z)-3-Ureidoacrylate peracid <-> (Z)-3-Peroxyaminoacrylate	264	0.000555298	166.75	0.000324111
9	RP01596	3.1.3.6	cpdB	Adenosine <-> 3-AMP	220	0.000448803	295	0.000631893

The results table shows that the purM gene is amongst the top ranked genes.

MNS for sequential data to identify sites and sequential order of metabolic regulation

MNS-data structure

The MNS-data structure for MNS inference on sequential data needs to have the following fields:

Field	Variable Type	Description
dataType	string	Tag describing the type of the input data. Options<br>*MetIdList* - metabolite annotation consist of m x 1 vector of metabolite ids<br>*fiaExp* - (internal use only) for fiaMiner annotation version 2.0<br>*fiaExp v3.0* - (internal use only) for fiaMiner annotation version 3.0
data	m x s double	Data matrix of metabolomics data quantifying sequential changes, e.g. log2(FC) comparing two consecutive time points; m = number of metabolites, s = number of sequence frames
annotation	m x 1 cell of string or struct	Metabolite annotation. For option MetIdList annotation is a m x 1 cell vector of metabolite ids. Internal: For option fiaExp annotation represents the annotation field of die fiaExp structure, for option fiaExp v3.0 annotation has the fields annotation and anndata that are the ones from the fiaExp

Initialization of model parameterization

The model parameterization initMNS can be initialized with

```
initMNS = mns_generateInitMNS('temporalModel', ParameterName, Value)
```

The initMNS structure has the same fields as for the univariate data. in addition the following fields are required

Field	Variable Type	Default Value	Description
tL3steps	double	40	Maximal number of scanning steps for sequential influence (lambda2).

For large metabolic models it is suggested to reduce the number of scanning steps through lambda1 and lambda2 to 20 and the number of neighborhood nodes to consider for LazyFlipper inference to 1. This can be done automatically using the *'temporalModel'* input parameter.

Run the MNS inference to identify sites and sequential order of regulation

The MNS inference for sequential data can be run using

```
mnsScanResults = mns_scanTime(metabolic_model, MNS_dataStruct, nameTag,
l1Range, l2Range, initMNS, plotResults)
```

Only the first two input arguments (metabolic_model, MNS_dataStruct) are required and need to be structured as described before. If the other variables are not defined they are set to the default values. Description of other Input arguments:

Input argument	Default	Description
nameTag	'temp'	Name of the folders in which the temporary data is stored.
l1Range	0	Scanning range of the lambda 1 variable (neighborhood influence parameter), e.g. [0:0.1:2]. If set to 0 (default), the range is determined automatically till nor more fractures are identified
L2Range	0	Scanning range of the lambda 2 variable (sequential influence parameter), e.g. [0:0.1:2]. If set to 0 (default), the range is determined automatically till nor more fractures are identified
plotResults	0	If 1 plots output results of the MNS scan

Functions to analyze the output of the MNS inference for sequential data

All functions for the analysis of the MNS inference for sequential data rely on a scoring function that aims to balance the amount of sequential and neighborhood fractures. The function is defined as

$$\begin{aligned} score(\lambda_1,\lambda_2,\boldsymbol{x}|w_t,w_n) = &\sum_{i=1}^{M} \psi_O\big(\hat{y}_i(\lambda_1,\lambda_2,x_i)\big) \\ &- w_s \frac{\#fractures_{sequence}\big(\hat{\boldsymbol{y}}(\lambda_1,\lambda_2,\boldsymbol{x})\big)}{\max\big(\#fractures_{sequence}\big)} \\ &- w_t \frac{\#fractures_{neighborhood}\big(\hat{\boldsymbol{y}}(\lambda_1,\lambda_2,\boldsymbol{x})\big)}{\max\big(\#fractures_{neighborhood}\big)}, \end{aligned}$$

where $\hat{\boldsymbol{y}}(\lambda_1,\lambda_2,\boldsymbol{x})$ is the optimal module label distribution derived by inference given λ_1, λ_2 and the observations $\mathbf{x}$, $\#fractures_{sequence}\big(\hat{\boldsymbol{y}}(\lambda_1,\lambda_2,\boldsymbol{x})\big)$ and $\#fractures_{neighborhood}\big(\hat{\boldsymbol{y}}(\lambda_1,\lambda_2,\boldsymbol{x})\big)$ are the sequence and neighborhood fracture counts given the inference solution $\hat{\boldsymbol{y}}$, $\max\big(\#fractures_{sequence}\big)$ and $\max\big(\#fractures_{neighborhood}\big)$ are the total count of possible fractures given the model structure, w_s and w_n are the weights determining the influence of the number of sequence and neighborhood fractures on the score function. This score function allows to balance the fracture frequency so that it is comparable between different experiments. By maximizing the score function the best combination $\hat{\lambda}_1,\hat{\lambda}_2$ can be derived with

$$\hat{\lambda}_1,\hat{\lambda}_2 = \underset{\lambda_1,\lambda_2}{\operatorname{argmax}}\big(score(\lambda_1,\lambda_2,\boldsymbol{x}|w_s,w_n)\big).$$

Generate overview of sites and sequential order of regulated reactions

```
[seqFracList, seqFracListOnlyFracPos, nFracList, nFracListSplit] ...
    = mns_scanTimeResults2table(mnsResults,metabolic_model,dWs,dWn,limit,
mode)
```

This function generates overview tables of the sites and timings of regulations. Overview of input arguments.

Input argument	Default	Required	Description
mnsResults	-	x	Result structure of the mns_scanTime function
metabolic_model	-	x	Metabolic Model
dWs	0.01		Step size of the increasing sequence influence weight ws
dWn	0.01		Step size of the increasing neighborhood influence weight wn
Limit	Inf		Maximal ws and wn. If inf, scans till no more fractures are found
Mode	'scan'		Mode of the results table. Options<br>• *'scan'* – lists fractures through scanning from ws = 0:dWs:limit and wn = 0:dWn:limit<br>• *'exact'* – list fractures at exact values of ws = dWs and wn = dWn defined by

Overview of output arguments

Output argument	Description
seqFracList	Overview of sequence fractures of metabolites ranked according to stability (ws) and length of the module label span around the fracture
seqFracListOnlyFracPos	Overview of sequence fractures of metabolites ranked according to stability (ws)
nFracList	Overview of neighborhood fractures
nFracListSplit	Overview of neighborhood fractures, splited into individual fractures

Plot fracture frequency and sum of observation potential

```
mns_plotFractureFrequency(mnsResults,metabolic_model,wsMax,wnMaxc,dWs,dWn)
```

This function plots the neighborhood and sequence fracture frequency as well as the sum of the observation potential with increasing neighborhood and sequence influence. The data is plotted in the weight range ws = 0:dWs:wsMax and wn = 0:dWn:wnMax. Overview of input arguments:

Input argument	Default	Required	Description
mnsResults	-	x	Result structure of the mns_scanTime function
metabolic_model	-	x	Metabolic Model
wsMax	-	x	Maximal value of the sequence influence weight ws
wnMax	-	x	Maximal value of the neighborhood influence weight wn
dWs		x	Step size of the increasing sequence influence weight ws
dWn		x	Step size of the increasing neighborhood influence weight wn

Plot score distributions for increasing weights ws and wn

```
mns_plotMultipleScreeningResults(mnsResults,metabolic_model,wsMax,wnMaxc,dWs
,dWn)
```

This function plots multiple lamba1 and lambda2 dependent score distributions with increasing weighting of neighborhood and sequential influence. The distributions are plotted in the weight range ws = 0:dWs:wsMax and wn = 0:dWn:wnMax. Overview of input arguments:

Input argument	Default	Required	Description
mnsResults	-	x	Result structure of the mns_scanTime function
metabolic_model	-	x	Metabolic Model
wsMax	-	x	Maximal value of the sequence influence weight ws
wnMax	-	x	Maximal value of the neighborhood influence weight wn
dWs		x	Step size of the increasing sequence influence weight ws
dWn		x	Step size of the increasing neighborhood influence weight wn

Plot score distribution and segmentation results for given weights ws and wn

```
[probArr, score, idx, idxScore] = mns_calcProbabilityScanData
(mnsResults,metabolic_model,ws,wn,plotDistributions,plotMnsResults)
```

This function plots multiple lamba1 and lambda2 dependent score distributions with increasing weighting of neighborhood and sequential influence. The distributions are plotted in the weight range ws = 0:dWs:wsMax and wn = 0:dWn:wnMax. Overview of input arguments:

Input argument	Default	Required	Description
mnsResults	-	x	Result structure of the mns_scanTime function
metabolic_model	-	x	Metabolic Model
ws	-	x	Value of the sequence influence weight ws
wn	-	x	Value of the neighborhood influence weight wn
plotDistributions		x	Plot fracture frequency, sum observation potential and score distributions dependent on lambda1 and lambda2 for given weights ws and wn
plotMnsResults		x	Plot MNS inference results for given weights ws and wn. These include Module label, sequence fracture and neighborhood fracture distributions.

Overview of output arguments:

Input argument	Description
probArr	Lambda1 and lambda2 dependent distribution of sum of observation potentials
score	Lambda1 and lambda2 dependent distribution of score
idx	Index of the MNS inference step with maximal score for given weights ws and wn
idxScore	Row and column indices of the maximal score in the score matrix for given weights ws and wn

Plot module labels and fractures of MNS inference results for range of weights ws and wn

```
mns_plotModuleLabelsAndFractures(mnsResults,metabolic_model,wsVec,wnVec)
```

This function plots MNS inference results, i.e. the module labels, sequence fractures and neighborhood fractures, for different values of ws and wn. Overview of input arguments:

Input argument	Default	Required	Description
mnsResults	-	x	Result structure of the mns_scanTime function
metabolic_model	-	x	Metabolic Model
wsVec	-	x	Vector of the sequence influence weights ws
wnVec	-	x	Vector of the neighborhood influence weight wn

Plot sequential metabolite data with module label overlay

```
mns_plotTcWithModules(mnsResults,MNS_dataStruct,metabolic_model,idx,
plotMode,metIdx)
```

This function plots MNS inference results, i.e. the module labels, sequence fractures and neighborhood fractures, for different values of ws and wn. Overview of input arguments:

Input argument	Default	Required	Description
mnsResults	-	x	Result structure of the mns_scanTime function
MNS_dataStruct	-	x	MNS data structure
metabolic_model	-	x	Metabolic Model
idx	-	x	Index of the MNS inference step with maximal score for given weights ws and wn obtained from *'mns_calcProbabilityScanData'*
plotMode	'area'		Type of the module overlay. Options:<br>• *'area'* – module labels are visualized as filled areas in the background, foreground shows sequential metabolite changes in black<br>• *'line'* – module labels are visualized as different colored segments of the line representing the metabolite changes
metIdx	1:allMetabolites		Model indices of the metabolites to be plotted. Default: all metabolites are plotted

Example 3: Identification of sites and sequential order of metabolic regulations in human fibroblasts treated with increasing concentrations of hydrogen peroxide

This example contains untargeted metabolomics data from fibroblasts treated with increasing concentrations of hydrogen peroxide (Data from Kuehne et al Mol Cell. 2015 Aug 6;59(3):359-71). We applied the algorithm on a data set and metabolic which was reduced to metabolites in pentose phosphate pathway, glycolysis and the citric acid cycle.

```
%% load the data
load('mns_example_sequential_mns_fibroblasts_H2O2 - WS.mat')
```

```
%% initialize mns
% go to mns folder and perform the execute command:
mns_initialize

%% perform automated coarse grained MNS inference
% initialize mns parameter settings
initMNS = mns_generateInitMNS('inferenceParameter',1,'nL1steps',20,
'tL3steps', 20);
initMNS.noOfclusters = 3;
initMNS.mean = [-0.10 0 0.10];
initMNS.meanType = 'fix';
initMNS.stdType = 'one group - all data';

model = KEGG_HSA_MNS_red;

% run the mns algorithm
mnsResultsFibroDill_coarse = mns_scanTime(model,dataStructFibroH2O2,
'fibro_dil_time_coarse',0,0,initMNS);

%% plot the fracture frequency and score distribution
% fracture frequency and sum of observation potential
mns_plotFractureFrequency(mnsResultsFibroDill_coarse,
model,0.25,0.25,0.01,0.01)
```

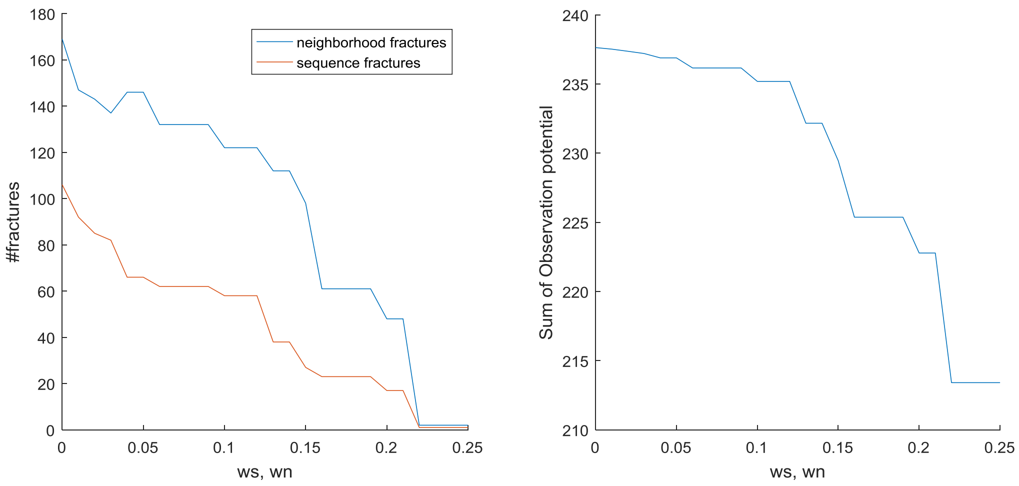


```
% Score distributions for increasing weights of ws and wt
mns_plotMultipleScreeningResults(mnsResultsFibroDill_coarse,
model,0.25,0.25,0.05,0.05)
```

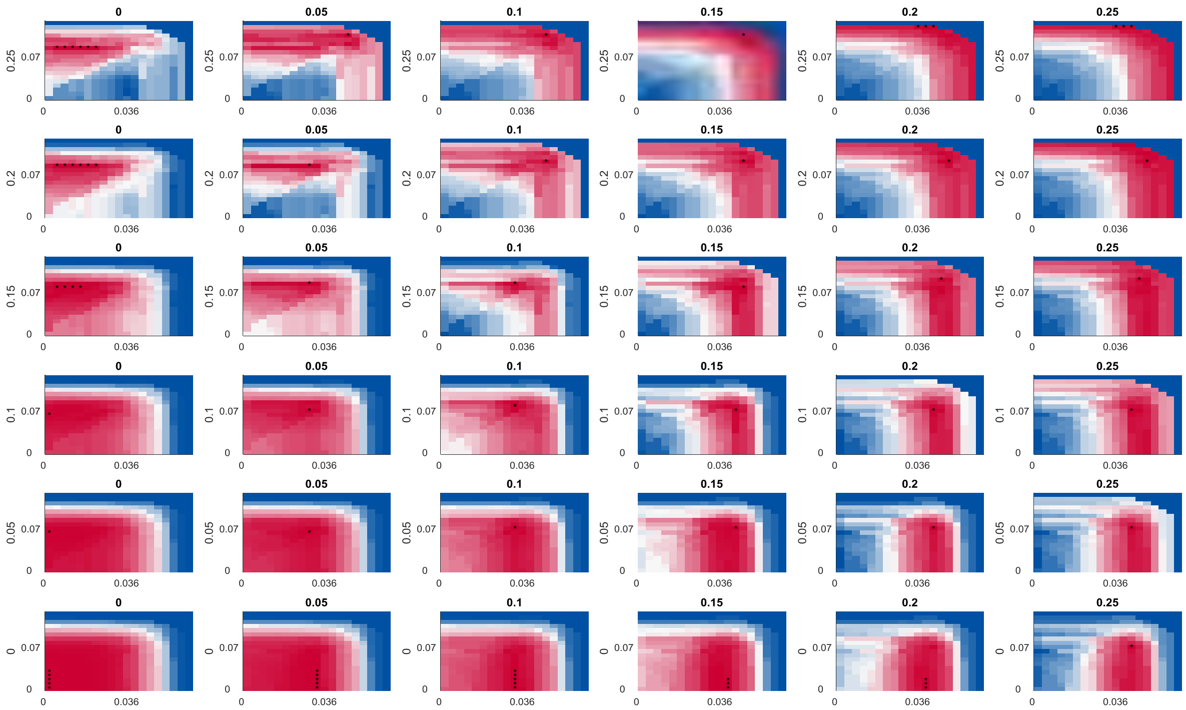

The score distribution and its maximum changes dependent on the weighting of the neighborhood and sequential influence ws and wn.

```
% Module label, sequence fracture and neighborhood fracture distribution
% for wn = ws = 0.0 (no influence)
mns_calcProbabilityScanData(mnsResultsFibroDill_coarse, model,0.0,0.0,
false);
```

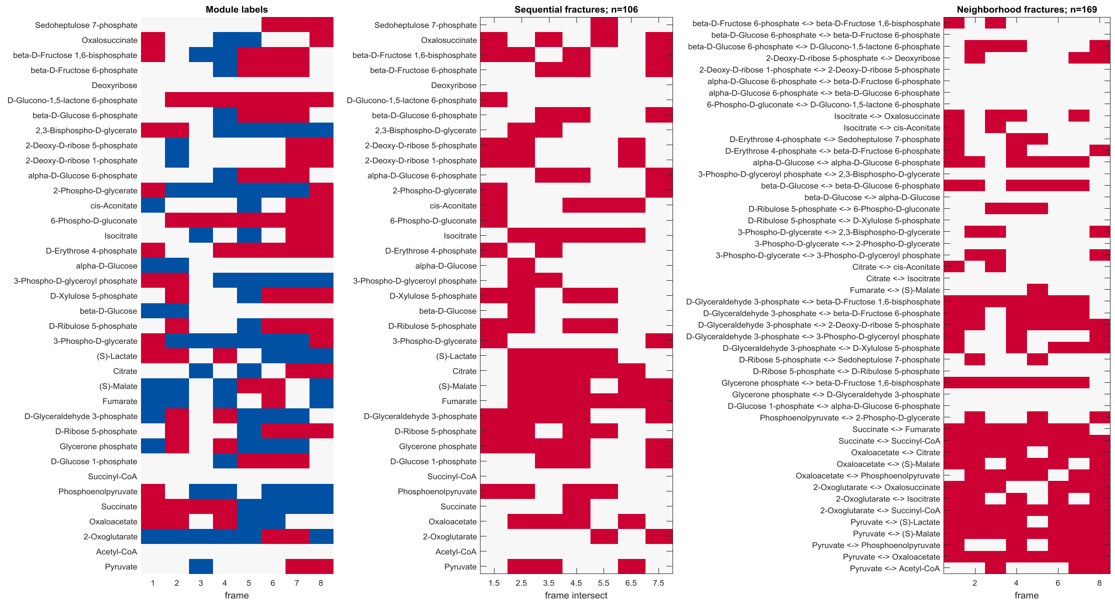


```
% Module label, sequence fracture and neighborhood fracture distribution
% for wn = ws = 0.21
```

```
[~,~,idx] = mns_calcProbabilityScanData(mnsResultsFibroDill_coarse,
model,0.21,0.21, false);

% Module label, sequence fracture and neighborhood fracture distribution
% for wn = ws = 0.25
mns_calcProbabilityScanData(mnsResultsFibroDill_coarse, model,0.25,0.25,
false);
```

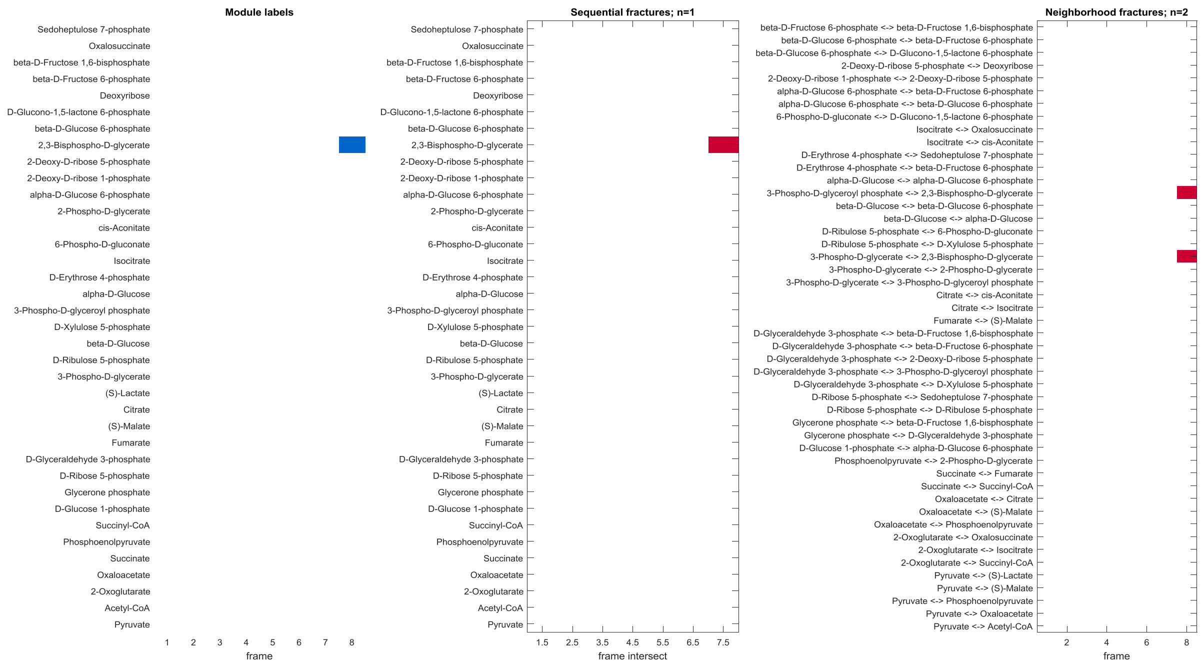

For w_s and w_t > 0.21 only one sequential and one neighborhood fracture is left which is biologically not relevant. Therefore we used ws = wn = 0.21 as reference point and investigated the fracture stability in a more fine grained scan thorugh lambda1 and lambda2 in the range from 0 to approximately double the maximal lambda value with the ws = wn = 0.

```
% Lambda values for ws = wn = 0.21;
disp(['lambda1 = ' num2str(mnsResultsFibroDill_coarse.nL1(idx))]);
>>lambda1 = 0.28928
disp(['lambda2 = ' num2str(mnsResultsFibroDill_coarse.tL3(idx))]);
>>lambda2 = 0.18003
```

In a next step we do a more fine grained scanning through the parameters lambda1 and lambda2 to identify relevant biological regulations

```
%% perform the MNS inference
% set the scanning range of lambda1 (nlRangeTemp) and lambda2 (tlRangeTemp)
% Note: the upperlimit of both lambda values have been determined manually
% before (see section before)
tlRangeTemp = [0 0.01:0.01:0.6];
nlRangeTemp = [0 0.01:0.01:0.6];
```

```
% initialize mns parameter settings
initMNS = mns_generateInitMNS('inferenceParameter',1,'nL1steps',20,
'tL3steps', 20);
initMNS.noOfclusters = 3;
initMNS.mean = [-0.10 0 0.10];
initMNS.meanType = 'fix';
initMNS.stdType = 'one group - all data';

% run the mns algorithm
mnsResultsFibroDill_fineRange = mns_scanTime(model,dataStructFibroH2O2,
'fibro_dil_time',nlRangeTemp,tlRangeTemp,initMNS);

%% Data analysis
% Sum of neighborhood fractures, sequential frame fracture and sum of
observation potential
mns_calcProbabilityScanData(mnsResultsFibroDill_fineRange,
model,0.00,0.00,true, false);
```

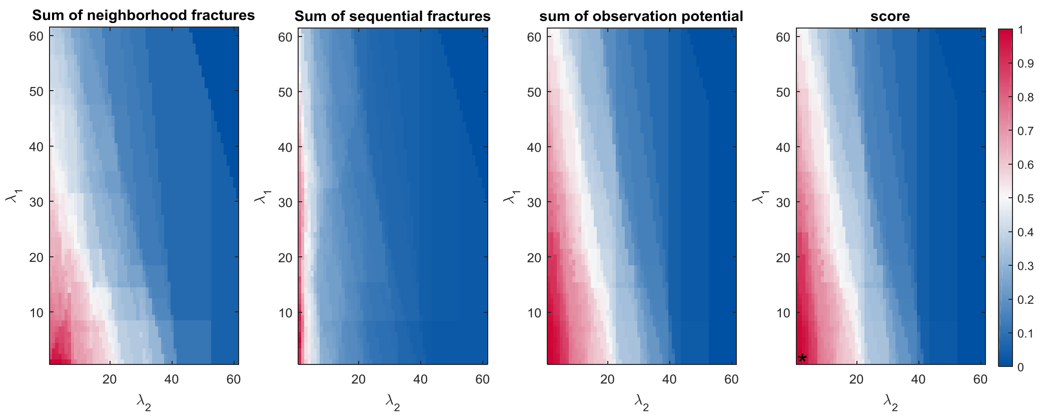


```
% Freqeuency of sequential frame and neighborhood fractures with increasing
% weights ws and wn
mns_plotFractureFrequency(mnsResultsFibroDill_fineRange,
model,0.5,0.5,0.01,0.01)
```

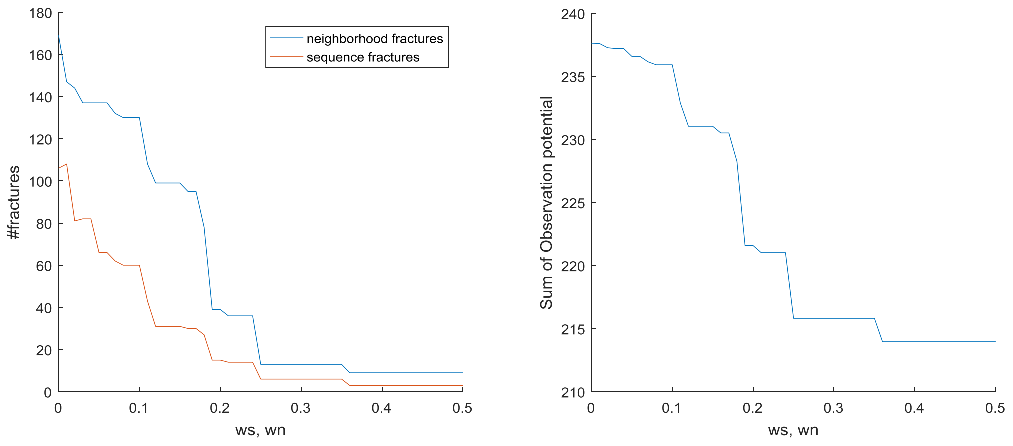

The number of fracture decrease with increasing weights ws and wn. At different ranges of ws and wn the number of fractures are at pseudo steady state, i.e. constant over a small range of weights.

```
% select the weights at which number of fractures are at pseudo
% steady state (i.e. constant over a small range of weights)
w = [0 0.05 0.1 0.15 0.24 0.3 0.4];

% Score distribution for increasing weights of ws and wn as defined before.
mns_plotMultipleScreeningResults(mnsResultsFibroDill_fineRange, model,w,w)
```

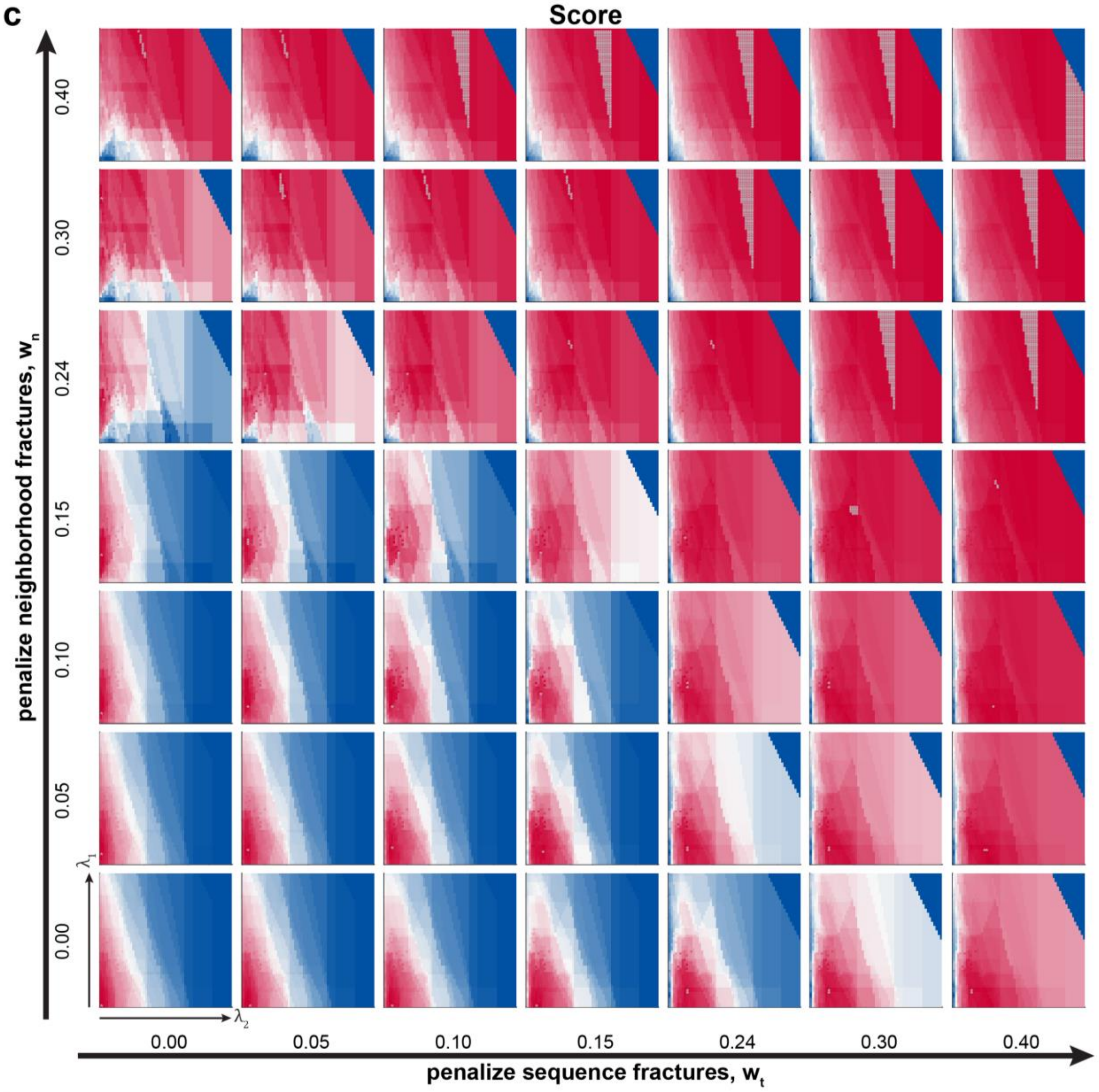


```
%Plot Module labels, sequence fractures and neighborhood fractures for all
metabolites and reactions with increasing weights ws and wn.
mns_plotModuleLabelsAndFractures(mnsResultsFibroDill_fineRange, model,w,w)
```

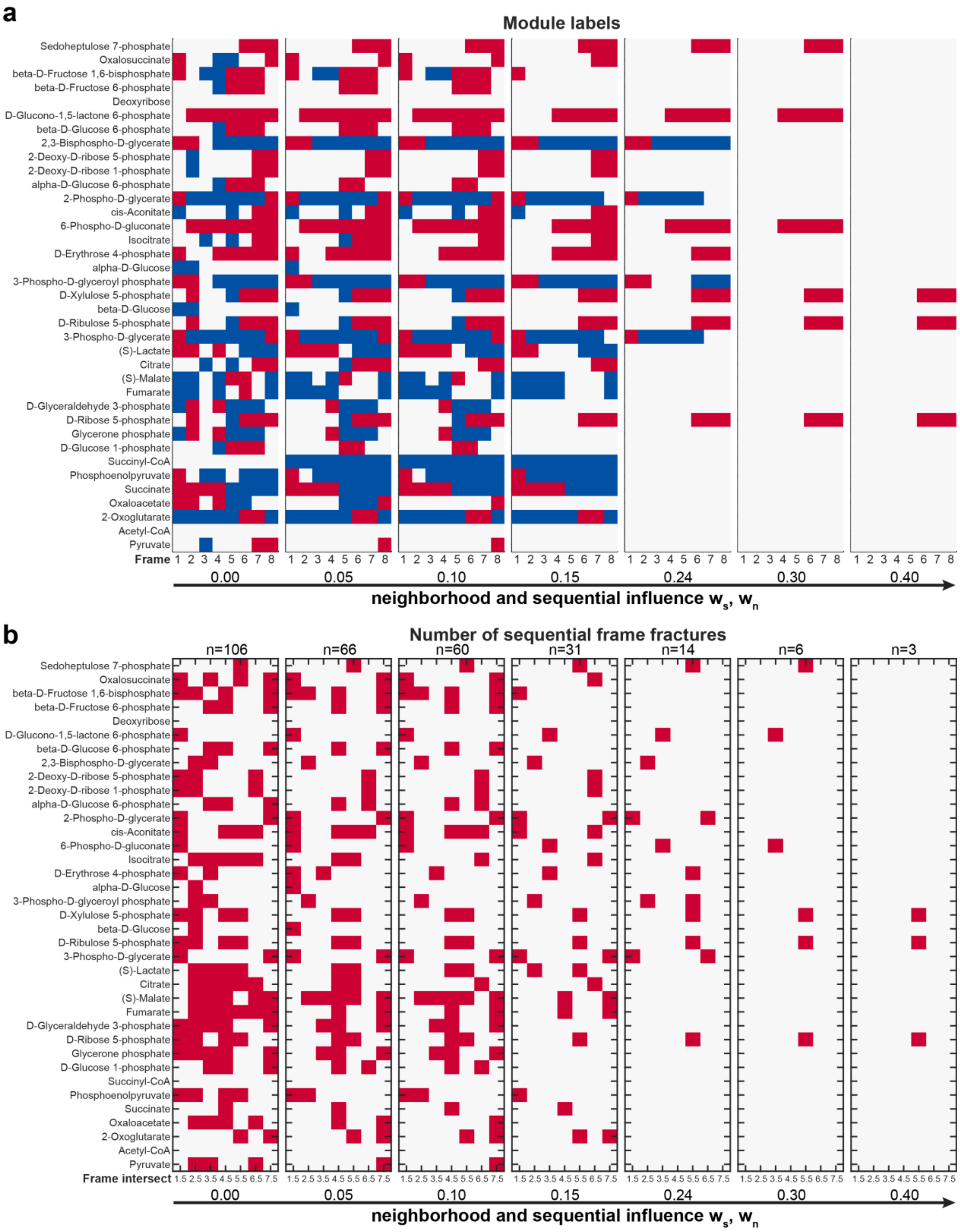
a
Module labels
Sedoheptulose 7-phosphate
Oxalosuccinate
beta-D-Fructose 1,6-bisphosphate
beta-D-Fructose 6-phosphate
Deoxyribose
D-Glucono-1,5-lactone 6-phosphate
beta-D-Glucose 6-phosphate
2,3-Bisphospho-D-glycerate
2-Deoxy-D-ribose 5-phosphate
2-Deoxy-D-ribose 1-phosphate
alpha-D-Glucose 6-phosphate
2-Phospho-D-glycerate
cis-Aconitate
6-Phospho-D-gluconate
Isocitrate
D-Erythrose 4-phosphate
alpha-D-Glucose
3-Phospho-D-glyceroyl phosphate
D-Xylulose 5-phosphate
beta-D-Glucose
D-Ribulose 5-phosphate
3-Phospho-D-glycerate
(S)-Lactate
Citrate
(S)-Malate
Fumarate
D-Glyceraldehyde 3-phosphate
D-Ribose 5-phosphate
Glycerone phosphate
D-Glucose 1-phosphate
Succinyl-CoA
Phosphoenolpyruvate
Succinate
Oxaloacetate
2-Oxoglutarate
Acetyl-CoA
Pyruvate
Frame 1 2 3 4 5 6 7 8
0.00 0.05 0.10 0.15 0.24 0.30 0.40
neighborhood and sequential influence w_s, w_n
b
Number of sequential frame fractures
n=106 n=66 n=60 n=31 n=14 n=6 n=3
Frame intersect 1.5 2.5 3.5 4.5 5.5 6.5 7.5
0.00 0.05 0.10 0.15 0.24 0.30 0.40
neighborhood and sequential influence w_s, w_n

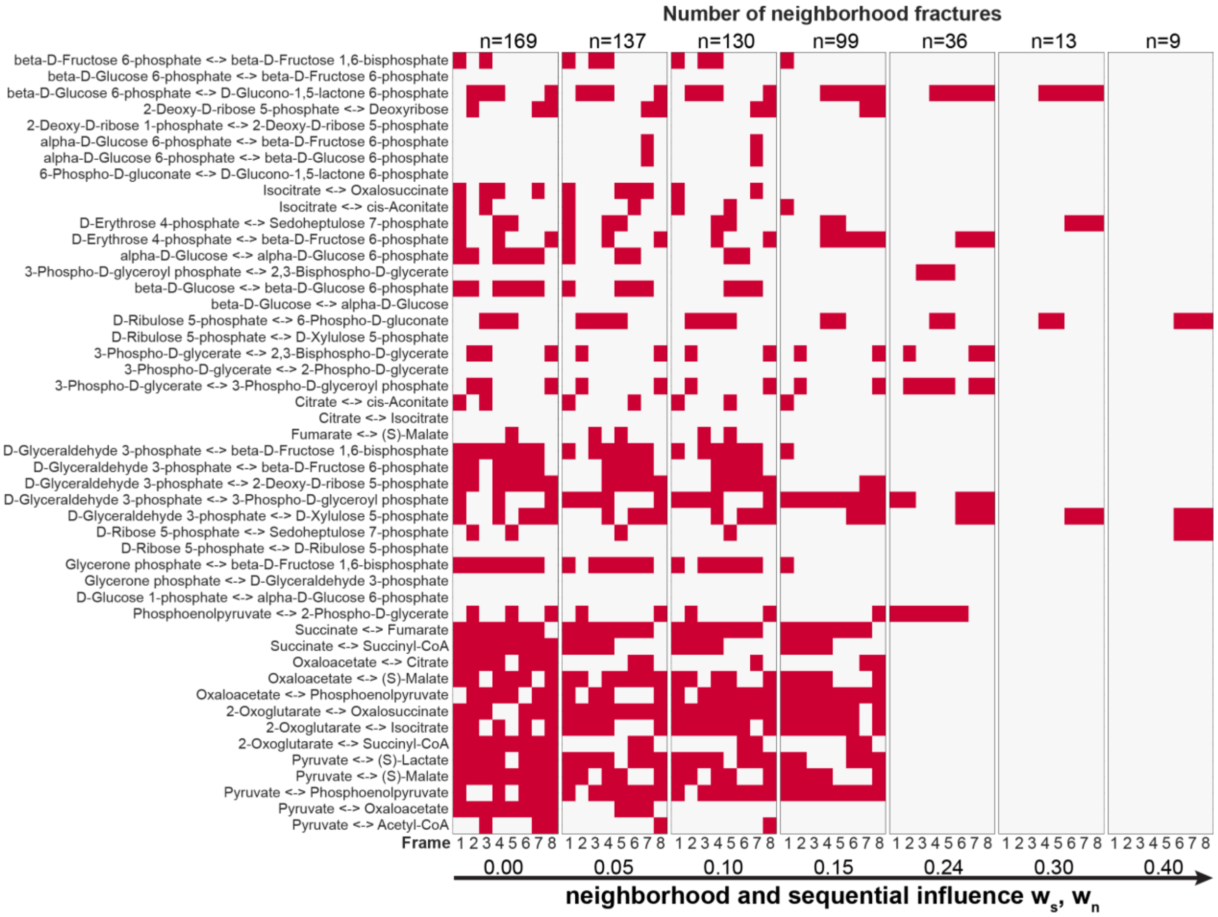

The analysis shows that with increasing weights ws and wn the module and fracture distributions are smoothed. Thereby relevant regulatory sites and timings are revealed.

```
% Application of MNS algorithm for sequential data on metabolomics data
% from fibroblasts treated with increasing concentrations of H2O2.

% left: sequence weight ws = 0, neighborhood weight wn = 0
[~,~,idxFibro] =
mns_calcProbabilityScanData(mnsResultsFibroDill_fineRange, model,0,0,
false, false);
mns_plotTcWithModules(mnsResultsFibroDill_fineRange, dataStructFibroH2O2,
model, idxFibro, 'area')

% middle: ws = 0.15, wn = 0.15
[~,~,idxFibro] =
mns_calcProbabilityScanData(mnsResultsFibroDill_fineRange, model,0.15,0.15,
false, false);
mns_plotTcWithModules(mnsResultsFibroDill_fineRange, dataStructFibroH2O2,
model, idxFibro, 'area')

% right: ws = 0.24, wn = 0.24
[~,~,idxFibro] =
mns_calcProbabilityScanData(mnsResultsFibroDill_fineRange, model,0.24,0.24,
false, false);
mns_plotTcWithModules(mnsResultsFibroDill_fineRange, dataStructFibroH2O2,
model, idxFibro, 'area')

% not shown: ws = 0.3, wn = 0.3
```

```
[~,~,idxFibro] =
mns_calcProbabilityScanData(mnsResultsFibroDill_fineRange, model,0.3,0.3,
false, false);
mns_plotTcWithModules(mnsResultsFibroDill_fineRange, dataStructFibroH2O2,
model, idxFibro, 'area')
```

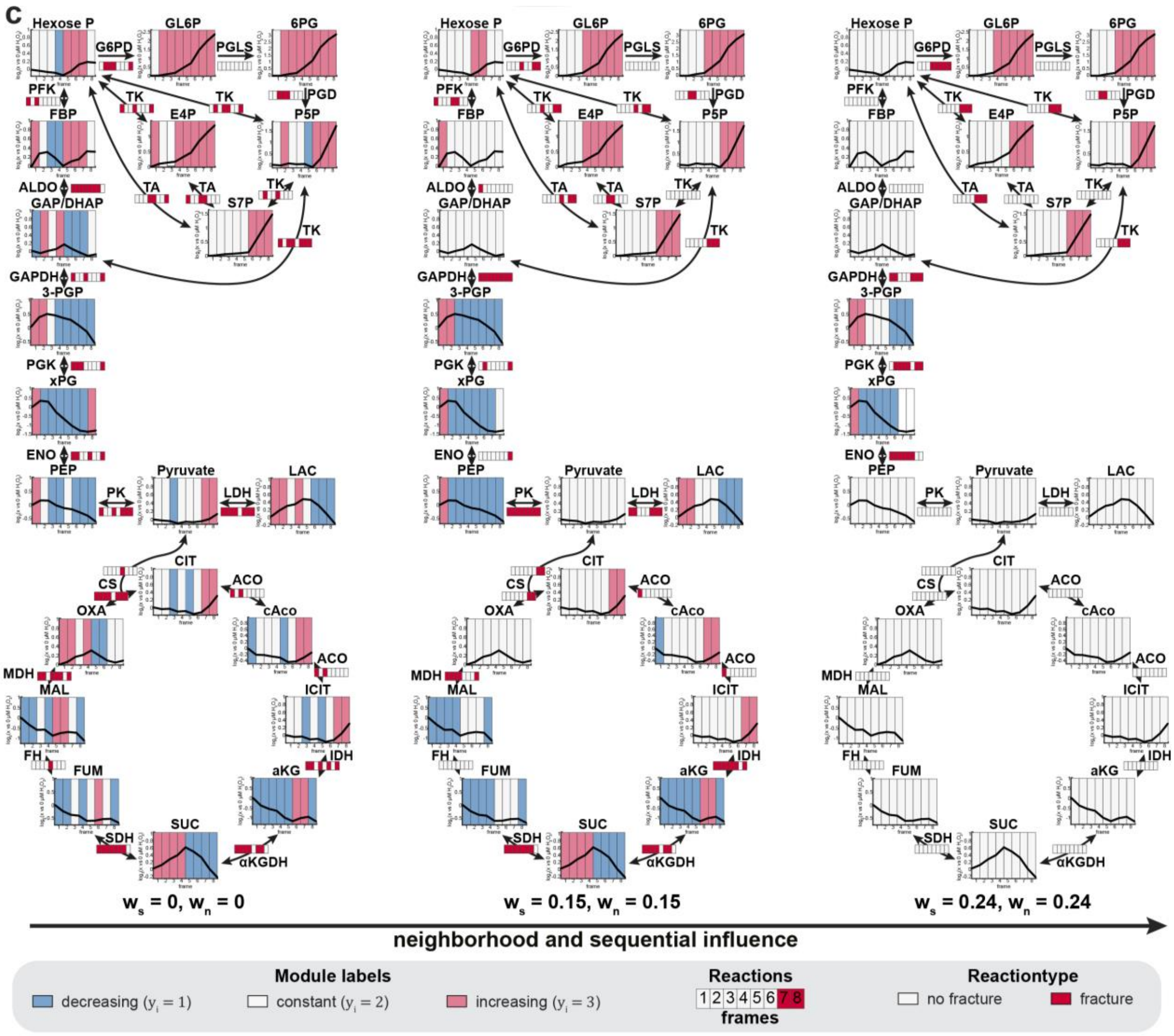

The analysis identifies relevant sites of metabolic regulations upon exposure to oxidative stress in upper glycolysis and the pentose phosphate pathway. Moreover the sequential order of regulations, first carbon flux gets rerouted into oxidative branch of the pentose phosphate pathway via activation G6PD, followed by a back flux into upper glycolysis via transketo (TK) and transaldolase (TA).

Copyright and License

Metabolic Network Segmentation Toolbox – A probabilistic graphical modelling tool to identify sites and sequential order of metabolic regulations

Copyright (C) 2016, Andreas Kühne & Nicola Zamboni

The Metabolic Network Segmentation Toolbox is free software: you can redistribute it and/or modify it under the terms of the GNU General Public License as published by the Free Software Foundation, either version 3 of the License, or any later version.

The Metabolic Network Segmentation Toolbox is distributed in the hope that it will be useful, but WITHOUT ANY WARRANTY; without even the implied warranty of MERCHANTABILITY or FITNESS FOR A PARTICULAR PURPOSE. See the GNU General Public License for more details.

You should have received a copy of the GNU General Public License along with this program. If not, see http://www.gnu.org/licenses/.

Appendix 1

Observation potential function type

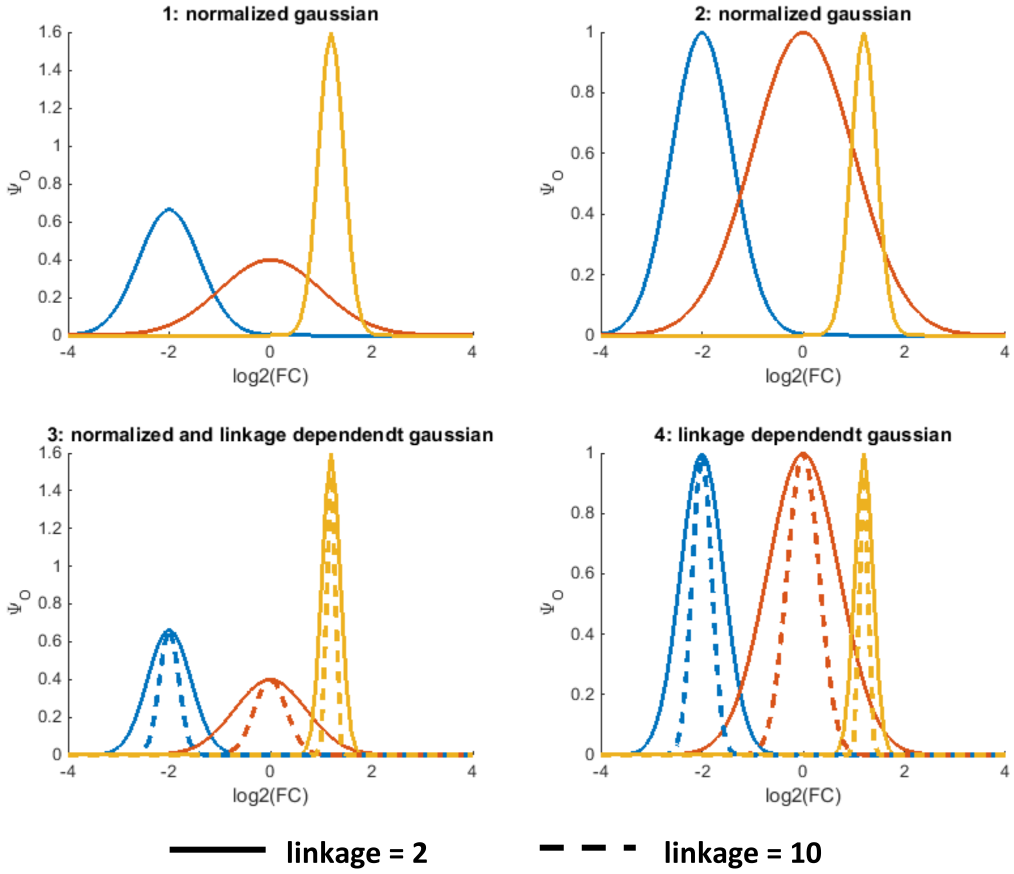

- Option 1: normalized gaussian

$$\psi_{O,i}(x_i, y_i) = \frac{\left(x_i - \mu(y_i)\right)^2}{\sqrt{2\pi} \cdot \sigma(y_i)} \cdot exp\left(-\frac{\left(x_i - \mu(y_i)\right)^2}{2\sigma(y_i)^2}\right)$$

- Option 2: not normalized gaussian

$$\psi_{O,i}(x_i, y_i) = exp\left(-\frac{\left(x_i - \mu(y_i)\right)^2}{2\sigma(y_i)^2}\right)$$

- Option 3: normalized Gaussian and linkage dependent gaussian

$$\psi_{O,i}(x_i, y_i) = \frac{\left(x_i - \mu(y_i)\right)^2}{\sqrt{2\pi} \cdot \sigma(y_i)} \cdot exp\left(-\text{nCliques}_i \cdot \frac{\left(x_i - \mu(y_i)\right)^2}{2\sigma(y_i)^2}\right)$$

- Option 4: not normalized Gaussian and linkage dependent gaussian

$$\psi_{O,i}(x_i, y_i) = exp\left(-\text{nCliques}_i \cdot \frac{\left(x_i - \mu(y_i)\right)^2}{2\sigma(y_i)^2}\right)$$

x_i = observed data of metabolite i
y_i = hidden state label of metabolite i
μ(yi) = hidden state dependent mean value
σ(yi) = hidden state dependent std value

nCliques$_i$ = number of cliques in which metabolite i is invovled

Neighborhood potential function type

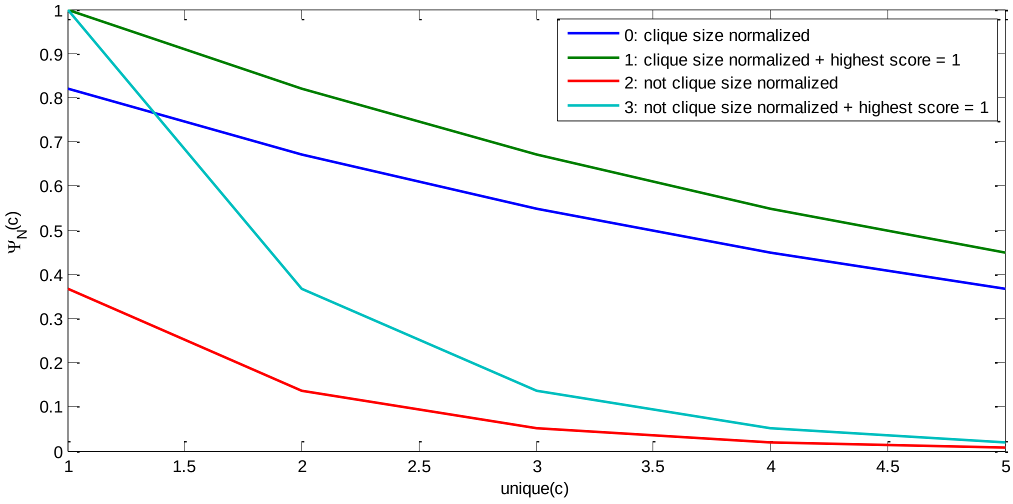

- Option 0: clique size normalized

$$\psi_N(c) = e^{-\lambda_{N,1}\frac{unique(c)}{size(c)}}$$

- Option 1: clique size normalized and highest score independent of clique size

$$\psi_N(c) = e^{-\lambda_{N,1}\frac{unique(c)-1}{size(c)}}$$

- Option 2: not clique size normalized

$$\psi_N(c) = e^{-\lambda_{N,1}\cdot\, unique(c)}$$

- Option 3: not clique size normalized and highest score independent of clique size

$$\psi_N(c) = e^{-\lambda_{N,1}\cdot\,(unique(c)-1)}$$
