# Supplementary figures and images for: Metaboverse enables automated discovery and visualization of diverse metabolic regulatory patterns
Source: Nat Cell Biol. 2023 Apr 3;25(4):616–25. doi: 10.1038/s41556-023-01117-9 (PMC10104781; doi:10.1038/s41556-023-01117-9)

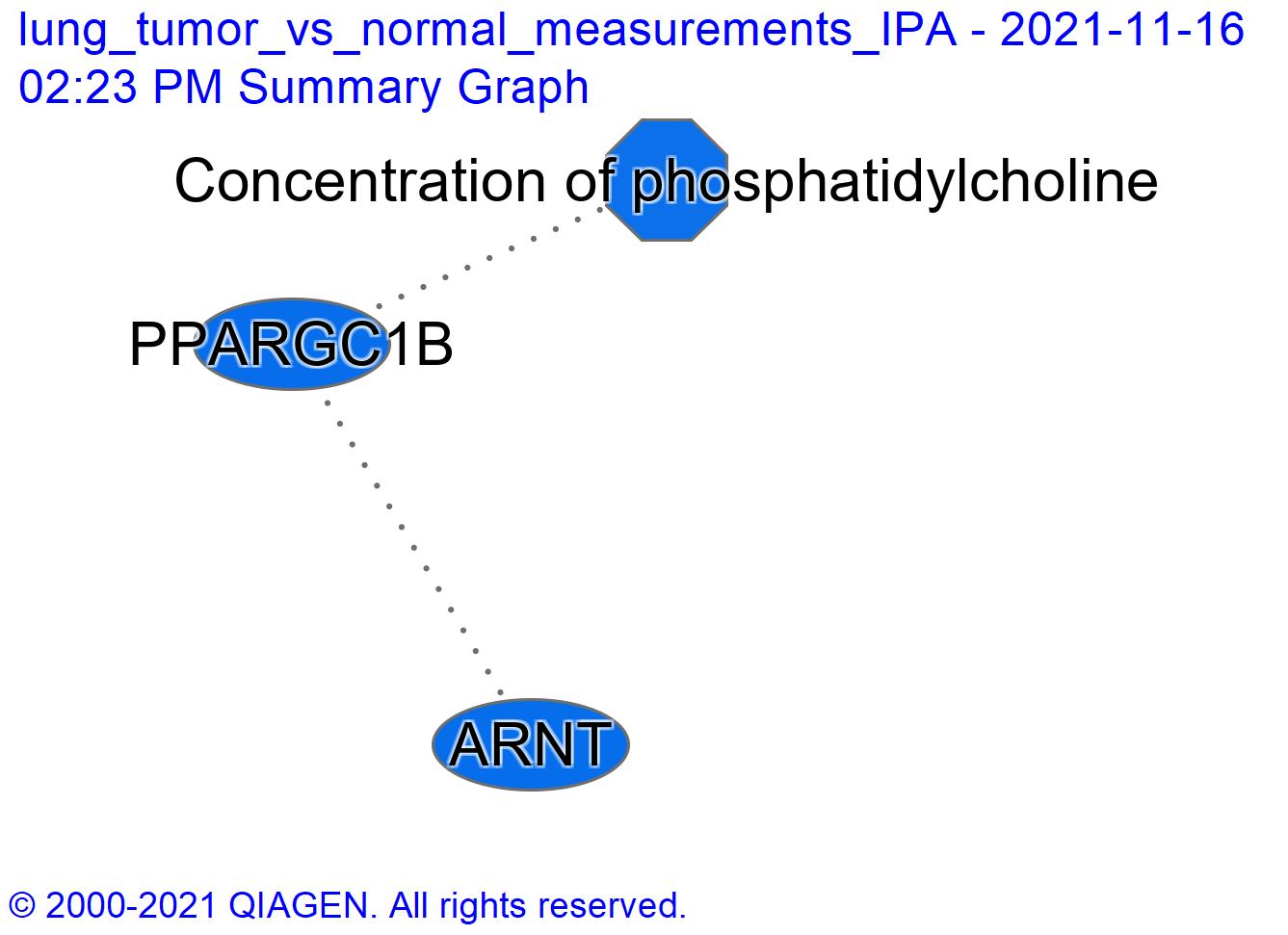

Supplement: Source Data Fig. 5 — Source numerical data. [file 41556_2023_1117_MOESM7_ESM.zip › fig_5_source/ipa/outputs/luad_ipa_graphical_summary.jpg]

Exposure 1

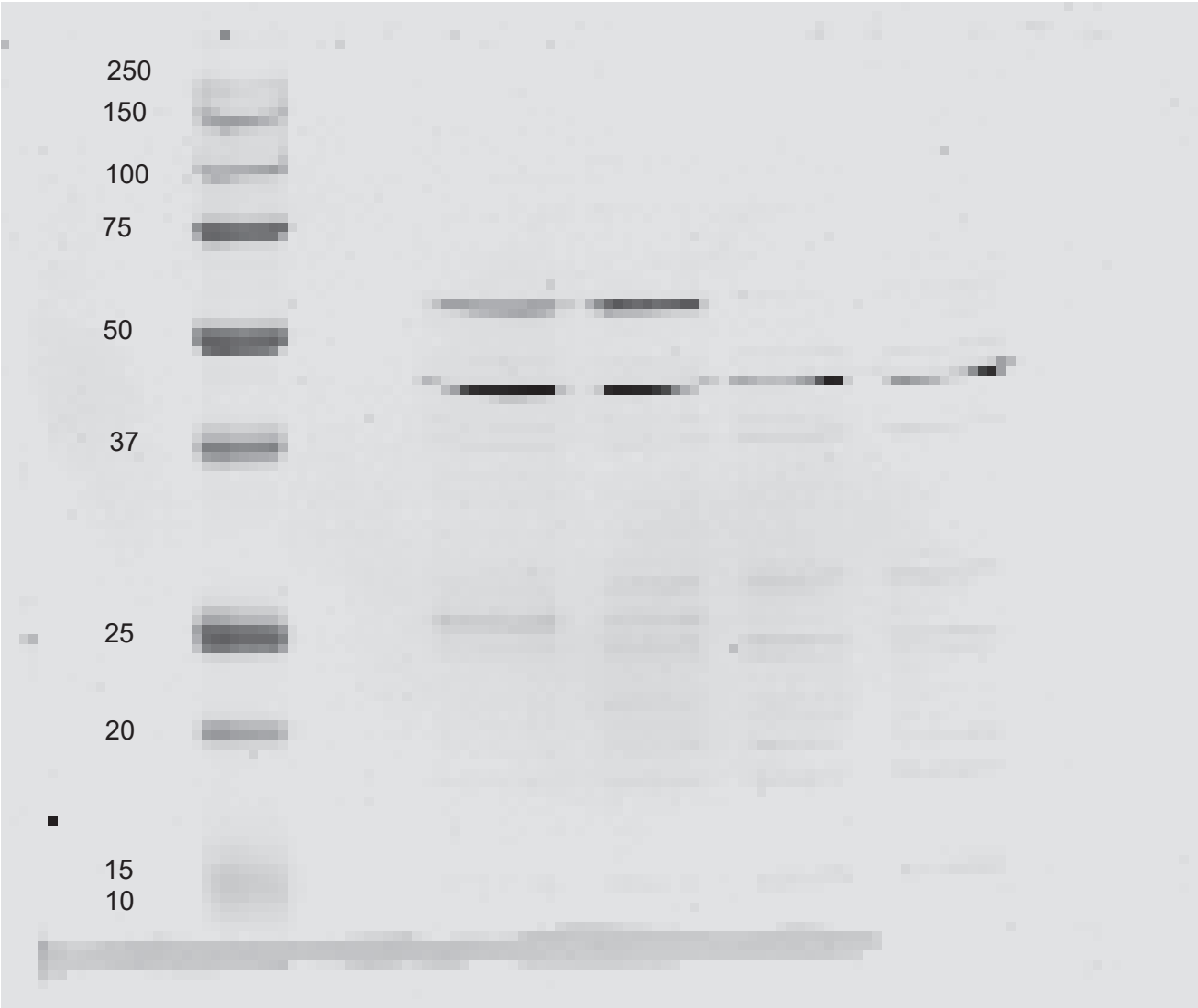

Exposure 2

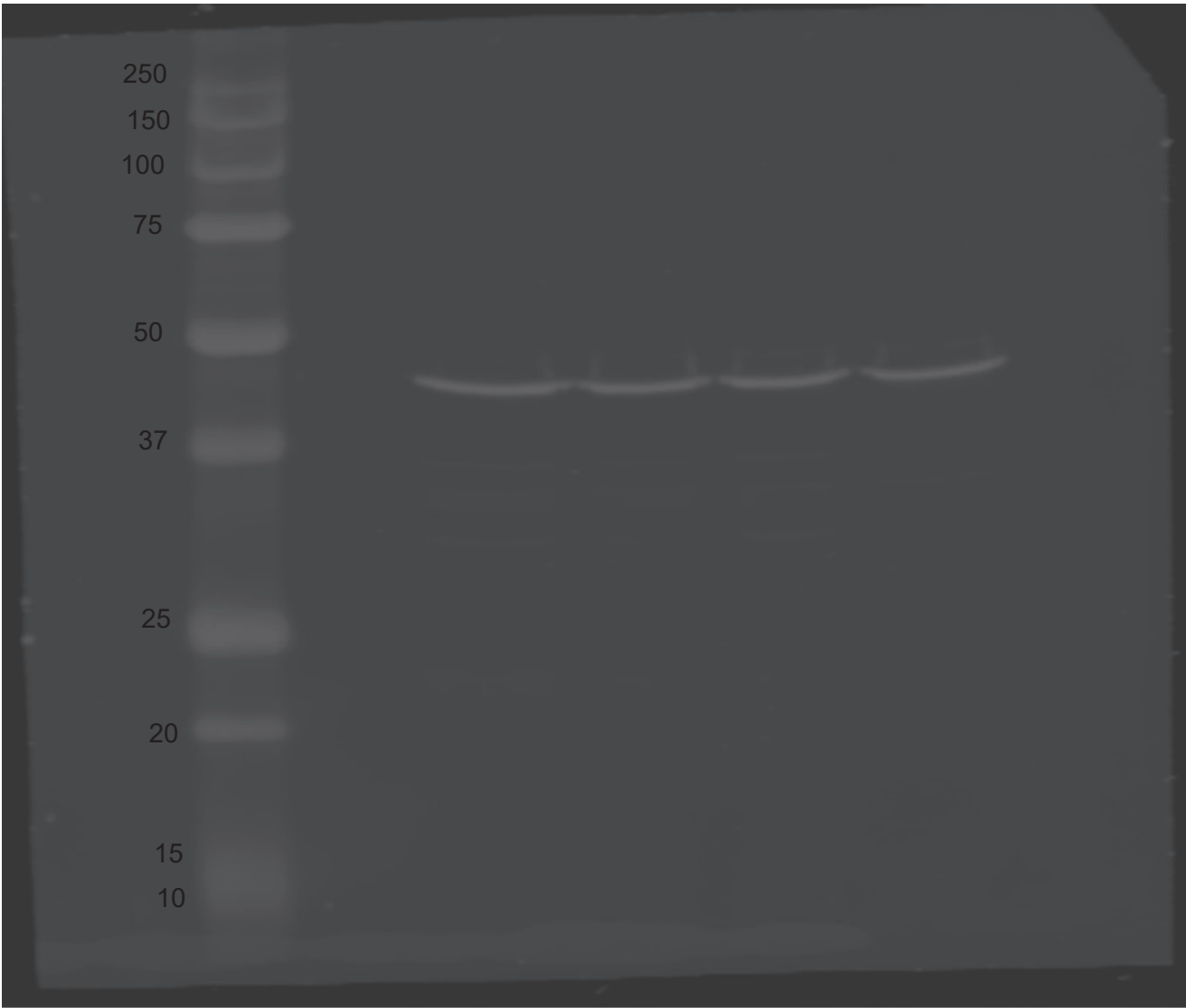

Supplement: Source Data Extended Data Fig./Table 10 — Source unprocessed blots, plate images and numerical data. [file 41556_2023_1117_MOESM11_ESM.pdf]
